# Associations of white potato intake and preparation methods with cardiometabolic health measures in US adults categorised by diabetes status

**DOI:** 10.1017/S0007114525106089

**Published:** 2026-04-14

**Authors:** Neda S. Akhavan, Susan N. Cheung, Bahram H. Arjmandi, Robert C. Hickner, Claire E. Berryman

**Affiliations:** 1 Department of Kinesiology and Nutrition Sciences, University of Nevada Las Vegas, Las Vegas, NV, USA; 2 Department of Health, Nutrition, and Food Sciences, Florida State University, Tallahassee, FL, USA; 3 Center for Advancing Exercise and Nutrition Research on Aging, Florida State University, Tallahassee, FL, USA; 4 Oak Ridge Institute for Science and Education, Belcamp, MD, USA; 5 Military Nutrition Division, US Army Research Institute of Environmental Medicine, Natick, MA, USA; 6 Pennington Biomedical Research Center, Louisiana State University, Baton Rouge, LA, USA

**Keywords:** National Health and Nutrition Examination Survey, Diabetes, Starchy carbohydrate, CVD, White potato

## Abstract

White potatoes are a major contributor to energy and nutrient intake in the USA, which supports investigating their relationship with cardiometabolic health. This cross-sectional analysis assessed relationships of total white potato intake and dietary patterns containing white potatoes prepared by various methods with markers of cardiometabolic health in adults categorised by diabetes status. The dietary intake assessment component of the National Health and Nutrition Examination Survey (2001–2018), What We Eat in America (WWEIA), was linked with the Food and Nutrient Database for Dietary Studies and Food Patterns Equivalents Database to rank the consumption of white potato-containing foods. Dietary patterns were determined by percent calories from white potatoes and main food groups in WWEIA using cluster analysis. Regression analysis assessed trends in individuals with (*n* 5467) and without (*n* 38 159) diagnosed diabetes. *P* < 0·01 was significant. The most consumed white potato-containing foods were French fries, potato chips and home fries. In adults without diagnosed diabetes, total white potato intake was positively associated with glucose, insulin, Homeostatic Model Assessment for Insulin Resistance and waist circumference. Glycated Hb was lower in those who primarily consumed dietary patterns with baked/boiled potatoes, and waist circumference was higher in those who primarily consumed dietary patterns with chips, fried potatoes or mashed potatoes compared with adults with no white potato intake. In adults without diagnosed diabetes, total white potato intake was associated with greater cardiometabolic risk, which may be due, in part, to frying as the predominate preparation method of white potatoes in the USA.

Over 29 million Americans have diagnosed diabetes, with the majority of cases being type 2 diabetes (T2D)^(1)^. T2D increases the risk of co-morbid micro- and macrovascular complications and mortality^(1,2)^. Following a healthy dietary intake pattern and engaging in physical activity are important behaviours for the prevention and management of T2D. There is a well-established inverse relationship between fruit and vegetable intake and the development and progression of T2D^(3,4)^. The 2020–2025 Dietary Guidelines for Americans recommends increasing fruit and vegetable intake since most Americans do not consume the recommended 1·5–2 cups of fruit and 2–3 cups of vegetables daily^(5)^. In addition, emphasis has been placed on both the quantity and quality of dietary carbohydrate intake for individuals with prediabetes and T2D^(6,7)^. Low-carbohydrate, low glycemic index (GI), Mediterranean and higher-protein diets have all been shown to improve glycemic control^(8)^. However, in a study by Wolever *et al.*^(9)^, patients with T2D who were randomised to consume a low GI, high GI or low-carbohydrate diet had no differences in glycated HbA1c concentrations following a 12-month dietary intervention, although postprandial glucose and C-reactive protein concentrations were lower on the low GI diet. These findings suggest high GI foods, in moderate amounts, may be safely incorporated into the diets of patients with T2D to provide variety and increase palatability.

In particular, white potatoes are a nutrient-dense vegetable containing vitamins (e.g. vitamin C), minerals (e.g. potassium), essential amino acids, fibre (e.g. resistant starch) and phytochemicals but are often avoided by patients with T2D due to their high starch content^(10,11)^. Several prospective cohort studies have reported an association between white potato intake and increased risk of T2D^(12–15)^. However, many of these observational studies did not account for cooking and preparation methods, which have important health implications^(16,17)^.

Preparation methods, cooking time and processing of potatoes (e.g. wet and dry heating, retrogradation) can affect resistant starch content, with baked potatoes retaining the highest levels^(18)^. Moreover, various cooking methods of potatoes such as baking, microwaving, boiling and frying, can affect the GI value and nutrient content of white potato-containing diets. Unfortunately, most preparation methods add a considerable amount of fat to white potatoes (e.g. fried white potatoes), which can have detrimental effects on health^(11)^. Preparation methods of white potatoes likely contribute, at least in part, to the observed associations between potato consumption and increased risk of T2D^(19,20)^. White potato intake and, more specifically, fried potato intake have been associated with an increased risk of T2D^(16,21)^. However, a moderate intake of boiled potatoes has been associated with a lower risk of developing T2D^(22)^.

The objective of this cross-sectional analysis was to assess relationships between total white potato intake or dietary intake patterns containing white potatoes prepared by various methods and diet quality and markers of cardiometabolic health in a nationally representative sample of individuals categorised by diabetes status using data from the National Health and Nutrition Examination Survey (NHANES) 2001–2018. To our knowledge, no study has assessed the relationship between white potato intake and cardiometabolic risk factors in individuals with diabetes, and few studies have examined the relationship between dietary patterns containing white potatoes and cardiometabolic risk in individuals with and without diabetes. We hypothesised that greater consumption of non-fried white potatoes would be associated with higher diet quality and better cardiometabolic health.

## Methods

### Study population

NHANES combines interviews and physical examinations of a nationally representative sample of the civilian, non-institutionalised US population to assess health and nutritional status. Currently, survey data are released every 2 years by the National Center for Health Statistics, which is part of the Centers for Disease Control and Prevention^(23)^. The Research Ethics Review Board at the National Center for Health Statistics approved the survey protocol, and all participants or proxies provided written informed consent. Detailed descriptions of the survey design and the data collection procedures are reported elsewhere^(23)^. Data analyses were obtained from NHANES 2001–2018 in participants ≥ 19years (*n* 43 626). Data were further categorised by diabetes status: no diabetes (*n* 38 159) or diagnosed with diabetes (*n* 5467) based on self-reported data (online Supplementary Fig. 1).

### Determination of white potato intake in the population

The dietary intake assessment component of NHANES, What We Eat in America (WWEIA), consists of 2, 24-h dietary recalls collected 3–10 d apart. The first 24-h recall is collected in-person and the second over the telephone by trained staff. WWEIA data are combined with the Food and Nutrient Database for Dietary Studies (FNDDS) to generate gram amounts and determine nutrient values. The Food Patterns Equivalents Database (FPED) is generated from foods and beverages in FNDDS, which are converted to the US Department of Agriculture (USDA) Food Patterns components. White potatoes, expressed as cup equivalents, are one of the thirty-seven USDA Food Patterns components. FPED includes Food Patterns equivalents for each food consumed by a respondent and total Food Patterns equivalents for each respondent in WWEIA. The total Food Patterns equivalents for each food consumed from day 1 dietary recalls were summed by USDA food code to determine population (NHANES 2001–2018) intakes of each white potato-containing food in cup equivalents. Population intakes were then ranked according to the most to least consumed white potato-containing foods.

### Determination of average intake of white potatoes

White potato intake (independent variable) was derived from the FPED for each respondent in WWEIA. The average intake of white potatoes was calculated using day 1 and day 2 dietary recalls. Participants were divided into quintiles based on white potato consumption, with quintile 1 containing all individuals with no white potato consumption.

### Determination of nutrient intake and diet quality

Nutrient intake data and food component data were derived from the USDA FNDDS and FPED, respectively, for each respondent in the WWEIA. Each nutrient and food component was averaged using day 1 and day 2 dietary recalls. Diet quality was determined using the Healthy Eating Index-2020 (HEI-2020), which measures individual adherence to the Dietary Guidelines for Americans^(24)^. Total HEI-2020 scores range from 0 to 100. Scores are calculated by summing thirteen components based on either adequacy or moderation. Adequacy components include total fruits, whole fruits, total vegetables, greens and beans, whole grains, dairy products, total protein foods, seafood and plant proteins and fatty acids. Moderation components include refined grains, sodium, added sugars and saturated fats. A higher HEI-2020 total score is associated with a better diet quality.

### Determination of dietary patterns

Dietary patterns were determined based on day 1 dietary recall mean percent calories from white potatoes and the fifteen main food groups in WWEIA and divided into clusters. Potato clusters were defined according to potato intake (no potato or potato intake) and primary potato preparation method (baked/boiled, chips, fried or mashed). The potato preparation methods were derived from the following WWEIA category codes in the FNDDS Main Food Descriptions dataset: 6802 white potatoes, baked or boiled; 5002 potato chips; 6804 French fries and other fried white potatoes; and 6806 mashed potatoes and white potato mixtures. Cluster 0 represents no white potato intake. Each preparation method was split into two clusters to maximise differences in each food group. For each generated cluster, the mean percent caloric intake by food group category is presented (Table 1).

**Table 1.** Potato dietary patterns of consumption (clusters) by potato preparation method and mean percent calories (kcal) within food groups in US adults with and without diagnosed diabetes Table 1 long description.

	Cluster	*n*	Potato	Dairy products	Protein foods	Mixed dishes	Refined grains	Whole grains	Snack/ sweets	Fats/oils	Fruit	Vegetable	Bev	Bev alcoholic
No diabetes														
No potato	0	24 166	0·00	6·87	15·51	23·19	10·70	3·58	14·27	3·60	3·17	2·58	9·60	4·28
Baked/boiled	1	988	9·14	6·75	23·95	3·13	9·83	3·80	14·44	4·91	3·36	3·63	9·04	5·09
	2	469	7·73	5·63	13·15	28·98	7·50	2·44	11·91	3·75	2·39	3·10	7·86	2·86
Chips	1	2635	9·16	7·08	16·53	9·42	11·22	2·73	15·09	3·63	2·48	2·04	12·07	6·01
	2	1371	8·52	4·79	9·73	37·98	8·31	2·50	10·24	2·96	1·89	1·11	7·83	2·04
Fried	1	3411	12·78	4·43	11·22	31·23	5·51	1·70	10·61	2·44	1·18	0·98	12·79	2·86
	2	2577	13·07	7·20	22·91	4·45	11·58	2·05	12·71	4·21	1·79	2·04	8·93	6·05
Mashed	1	1051	10·22	4·62	13·98	30·07	6·12	1·79	12·76	3·21	1·74	2·23	6·97	4·12
	2	2437	12·32	6·05	20·76	4·90	10·16	2·67	16·16	3·86	2·62	3·41	10·25	4·03
Diagnosed with diabetes														
No potato	0	3481	0·00	7·29	17·81	22·13	12·80	4·84	13·53	3·81	4·11	3·05	6·55	2·29
Baked/boiled	1	38	5·85	4·70	10·40	49·44	4·16	4·02	6·26	1·40	1·25	2·38	7·74	1·29
	2	210	9·38	6·48	22·53	7·15	14·99	4·45	12·65	5·30	4·13	4·36	5·88	1·18
Chips	1	255	8·33	6·89	8·69	30·14	8·91	4·34	13·60	3·51	2·31	2·00	7·58	1·73
	2	247	11·50	5·63	27·25	6·68	14·36	3·71	10·20	3·84	2·74	2·33	6·58	2·89
Fried	1	493	13·37	5·67	25·66	9·83	10·91	3·26	10·80	4·21	2·38	2·61	5·70	3·21
	2	223	15·23	3·39	6·25	40·32	6·49	1·83	11·40	2·22	2·03	0·79	7·47	0·68
Mashed	1	353	12·90	5·70	24·31	3·34	10·79	4·88	16·96	4·49	3·20	3·50	6·57	0·95
	2	167	11·14	5·04	13·64	28·66	8·40	3·51	8·18	3·47	3·15	3·52	6·67	2·84

Data from Day 1 recall from NHANES 2001-2018. Proc Cluster of SAS was used to define clusters. What We Eat In America food groups were used to develop clusters based on percent calories from each food group with potatoes by preparation method as the centroid of the cluster. Cluster 0 represents no potato intake. NHANES; National Health and Nutrition Examination Survey; Bev, beverage. Bolded text represents a significant p-value (p < 0.01).

### Cardiometabolic risk factors

The following cardiometabolic risk factors (dependent variables) were extracted from examination and laboratory data files: glucose (mg/dl), glycated Hb (%), insulin (μU/ml), systolic blood pressure (BP) (mmHg), diastolic BP (mmHg), total cholesterol (mg/dl), LDL-cholesterol (mg/dl), HDL-cholesterol (mg/dl), TAG (mg/dl) and waist circumference (cm). HOMA-IR was calculated as the product of fasting insulin (µU/ml) and fasting glucose (mmol/l) divided by 22·5^(25)^.

### Covariates

Nutrient intake and HEI-2020 total and component scores according to usual white potato intake in US adults were adjusted for self-reported gender (male or female), race/ethnicity (Mexican American, other Hispanic race, non-Hispanic White, non-Hispanic Black or other race), age (years), smoking status (current smoker, former smoker, never smoked), alcohol use (number of alcoholic drinks per day over last 12 months), physical activity (sedentary, moderate or vigorous) and monthly poverty:income ratio index. All cardiometabolic dependent variables in the total white potato analysis were adjusted for self-reported gender (male or female), race/ethnicity (Mexican American, other Hispanic race, non-Hispanic White, non-Hispanic Black or other race), age (years), smoking status (current smoker, former smoker, never smoked), alcohol use (number of alcoholic drinks per day over last 12 months), physical activity (sedentary, moderate or vigorous) and monthly poverty:income ratio index in model 1. Model 2 adjusted for all the variables in model 1 and total energy intake (kcal/d). Model 3 adjusted for all the variables in model 2 and saturated fat (g/d) and sodium (mg/d). Model 3 adjusted for saturated fat and sodium to account for ingredients commonly added to potatoes that are high in these nutrients. Model 4 (primary statistical model) adjusted for all the variables in model 3 and carbohydrate intake (g/d). Model 5 adjusted for all the variables in model 4 and BMI (for all non-weight-related variables). Model 6 adjusted for all the variables in model 2 and relative intake of saturated fat (g/1000 kcal) and sodium (mg/1000 kcal) intake. Model 7 adjusted for all the variables in model 6 and relative carbohydrate intake (g/1000 kcal). Model 8 adjusted for all the variables in model 7 and BMI (for all non-weight-related variables). For the dietary pattern cluster analyses, cardiometabolic risk factors were adjusted with model 2 covariates, and HEI-2020 total and component scores were adjusted with model 1 covariates.

### Statistical analyses

Linear regression was used to assess associations between demographic characteristics, nutrient intake, total and component HEI scores, cardiometabolic risk factors (dependent variables) and white potato intake (independent variable), as either a continuous variable (linear trend) or a categorical variable (quintile trend), after adjustment for covariates. Least-squares means and standard errors of demographic variables, average nutrient intake variables, total and component HEI scores and cardiometabolic risk factors are reported for each white potato intake quintile. In addition, regression analyses were used to assess associations between cardiometabolic risk factors and HEI-2020 total and component scores (dependent variables) and white potato-containing dietary patterns (cluster 0, 1 or 2; independent variable) by white potato preparation method, after adjustment for covariates. Least-squares means and standard errors of cardiometabolic risk factors, as well as the HEI-2020 total and component scores, are reported for each cluster. The normality of the variables was assessed by examining skewness and kurtosis and visually inspecting distribution and probability plots. If variables were non-normally distributed, they were log-transformed for analysis. For log-transformed variables, *P*-values are derived from the models in which the dependent variable was log-transformed; means, SE and beta values are derived from the non-transformed data. Data were analysed using SAS v9.4 (Research Triangle Institute). Appropriate weighting factors were used to adjust for oversampling of selected groups, survey non-responses of some individuals and the day of the week on which the interview was conducted. To account for multiple comparisons and the large sample size, a more conservative significance level of *P* < 0·01 was used to reduce the risk of type I error.

## Results

In both adults with no diabetes (*n* 38 159) and adults with diabetes (*n* 5467), French fries (fast food), potato chips and home fries were the top three consumed white potato-containing foods in the USA (online Supplementary Table 1). Baked white potato with nothing added and the peel eaten was the 29th most consumed form of white potato in adults with no diabetes. In adults diagnosed with diabetes, a baked potato with nothing added and the peel eaten was not in the top 100 most consumed white potato-containing foods.

In adults without diabetes, white potato intake was inversely related to (linear trends) being female, Mexican American, other Hispanic and other race(s) and positively associated with being non-Hispanic white and a current smoker (online Supplementary Table 2). In adults with diagnosed diabetes, white potato intake was inversely related to (linear trends) being female, Mexican American, non-Hispanic Black and other race(s) and having a poverty:income ratio of 1·35–1·85 and positively associated with being non-Hispanic white and having a poverty:income ratio > 1·85. There were positive associations for white potato intake with absolute energy, macronutrient, cholesterol, sodium and potassium intake in those with and without diabetes (online Supplementary Table 3). When nutrient intake was adjusted for energy intake (i.e. per 1000 kcal), there were negative associations (linear trends) between white potato intake and carbohydrate, protein, fibre and total sugar intake and positive associations between white potato intake and total fat, polyunsaturated fat, monounsaturated fat, saturated fat and potassium intake in those without diabetes (online Supplementary Table 4). In those with diabetes, there were negative associations (linear trends) between white potato intake and energy-adjusted protein and total sugar intake and positive associations between white potato intake and energy-adjusted total fat, polyunsaturated fat, monounsaturated fat, saturated fat and potassium intake (online Supplementary Table 4). Greater white potato intake was associated with a greater total vegetable HEI score but with a lower total HEI score (quintile trend only) in adults without diagnosed diabetes (online Supplementary Table 5). In adults with diagnosed diabetes, greater white potato intake was associated with a greater total vegetable HEI score and a greater total HEI score.

In adults without diabetes, greater white potato intake was positively associated (linear trends) with glucose for models 2, 3 and 4, waist circumference for models 1 and 2 and insulin, HOMA-IR and diastolic BP for model 1 (Table 2). Total white potato intake was positively associated (quintile trends) with insulin concentrations, HOMA-IR and waist circumference (models 1–4) in adults without diabetes. When model 4 was further adjusted for BMI (model 5) or models were adjusted for relative nutrient intake (i.e. nutrients per 1000 kcal; models 6–8) instead of absolute nutrient intake, associations between white potato intake and glucose, insulin and HOMA-IR were no longer significant. However, the quintile trend relationship between white potato intake and waist circumference remained significant even after adjustment for relative nutrient intake (models 6 and 7; online Supplementary Table 6). In adults with diabetes, there were no significant relationships between white potato intake and cardiometabolic outcomes (Table 3 and online Supplementary Table 6).

**Table 2.** Associations of average white potato intake with cardiometabolic risk factors in US adults without diabetes: NHANES 2001–2018 Table 2 long description.

			Quintiles of average white potato intake (cup eq)										Quintile trend			Linear trend		
	*n*	Model	1		2		3		4		5		*β*	se	*P*	*β*	se	*P*
			Mean	se	Mean	se	Mean	se	Mean	se	Mean	se						
Glucose, mg/dl^1^	17 071	1	102·4	0·4	102·2	0·4	103·1	0·6	102·5	0·5	103·6	0·6	0·24	0·12	0·0428	0·95	0·39	0·0112
		2	102·4	0·4	102·2	0·4	103·1	0·6	102·5	0·5	103·7	0·6	0·25	0·12	0·0332	1·00	0·39	**0·0081**
		3	102·5	0·4	102·3	0·4	103·1	0·6	102·6	0·5	103·7	0·6	0·24	0·12	0·0403	0·97	0·39	**0·0092**
		4	102·5	0·4	102·3	0·4	103·1	0·6	102·6	0·5	103·7	0·6	0·24	0·12	0·0403	0·96	0·39	**0·0093**
Glycated Hb, %^1^	37 491	1	5·56	0·01	5·55	0·01	5·57	0·01	5·56	0·01	5·59	0·01	0·01	0·00	0·0387	0·02	0·01	0·0777
		2	5·56	0·01	5·55	0·01	5·57	0·01	5·56	0·01	5·58	0·01	0·00	0·00	0·1992	0·01	0·01	0·3395
		3	5·57	0·01	5·55	0·01	5·57	0·01	5·56	0·01	5·58	0·01	0·00	0·00	0·2536	0·01	0·01	0·4300
		4	5·56	0·01	5·55	0·01	5·57	0·01	5·56	0·01	5·58	0·01	0·00	0·00	0·2554	0·01	0·01	0·4434
Insulin, μU/ml^1^	16 748	1	11·5	0·2	11·3	0·3	12·7	0·4	12·5	0·4	12·2	0·3	0·25	0·07	**0·0003**	0·52	0·20	**0·0063**
		2	11·6	0·2	11·3	0·3	12·7	0·4	12·5	0·4	12·1	0·3	0·22	0·07	**0·0016**	0·39	0·21	0·0278
		3	11·6	0·2	11·4	0·3	12·7	0·4	12·5	0·4	12·1	0·3	0·21	0·07	**0·0021**	0·37	0·20	0·0325
		4	11·6	0·2	11·4	0·3	12·7	0·4	12·5	0·4	12·1	0·3	0·21	0·07	**0·0022**	0·36	0·20	0·0362
HOMA-IR^1^	16 748	1	3·04	0·07	2·97	0·08	3·38	0·12	3·28	0·11	3·26	0·09	0·07	0·02	**0·0003**	0·16	0·06	**0·0040**
		2	3·06	0·07	2·98	0·08	3·38	0·12	3·28	0·11	3·22	0·09	0·06	0·02	**0·0013**	0·13	0·06	0·0154
		3	3·07	0·07	3·00	0·08	3·39	0·12	3·29	0·11	3·23	0·09	0·06	0·02	**0·0018**	0·12	0·06	0·0181
		4	3·07	0·07	3·00	0·08	3·39	0·12	3·29	0·11	3·23	0·09	0·06	0·02	**0·0019**	0·12	0·06	0·0201
Systolic BP, mmHg	37 885	1	124·4	0·3	124·0	0·5	124·5	0·4	125·0	0·4	125·0	0·4	0·18	0·09	0·0492	0·61	0·29	0·0382
		2	124·4	0·3	124·0	0·5	124·5	0·4	125·0	0·4	124·9	0·4	0·16	0·09	0·0797	0·56	0·29	0·0595
		3	124·4	0·3	124·0	0·5	124·5	0·4	125·0	0·4	124·9	0·4	0·16	0·09	0·0786	0·55	0·29	0·0597
		4	124·4	0·3	124·0	0·5	124·5	0·4	125·0	0·4	124·9	0·4	0·16	0·09	0·0780	0·56	0·29	0·0585
Diastolic BP, mmHg	37 741	1	71·4	0·2	70·6	0·3	71·3	0·3	72·0	0·3	71·9	0·3	0·16	0·06	0·0127	0·53	0·18	**0·0049**
		2	71·5	0·2	70·7	0·3	71·3	0·3	72·0	0·3	71·7	0·3	0·11	0·06	0·1001	0·35	0·19	0·0641
		3	71·5	0·2	70·7	0·3	71·3	0·3	72·0	0·3	71·7	0·3	0·10	0·06	0·1103	0·34	0·19	0·0720
		4	71·5	0·2	70·7	0·3	71·3	0·3	72·0	0·3	71·7	0·3	0·10	0·06	0·1097	0·34	0·19	0·0701
Total cholesterol, mg/dl	37 124	1	197·4	0·8	196·5	1·0	196·6	1·1	197·8	1·0	197·1	1·1	–0·02	0·22	0·9331	0·24	0·77	0·7540
		2	197·4	0·8	196·5	1·0	196·6	1·1	197·8	1·0	197·2	1·1	0·01	0·22	0·9743	0·34	0·78	0·6644
		3	197·4	0·8	196·5	1·0	196·6	1·1	197·8	1·0	197·1	1·1	–0·02	0·22	0·9408	0·28	0·78	0·7234
		4	197·5	0·8	196·6	1·0	196·6	1·1	197·8	1·0	197·2	1·1	–0·01	0·22	0·9468	0·30	0·78	0·6997
LDL-cholesterol, mg/dl	16 635	1	119·1	0·8	116·0	1·0	117·4	1·2	117·6	1·2	115·8	1·3	–0·64	0·26	0·0176	–1·74	0·80	0·0308
		2	119·0	0·8	115·9	1·1	117·4	1·2	117·7	1·2	116·1	1·3	–0·56	0·27	0·0391	–1·49	0·82	0·0732
		3	119·0	0·8	116·0	1·1	117·4	1·2	117·7	1·2	116·1	1·3	–0·57	0·27	0·0348	–1·51	0·83	0·0712
		4	119·0	0·8	116·0	1·1	117·4	1·2	117·7	1·2	116·1	1·3	–0·57	0·27	0·0356	–1·49	0·83	0·0737
HDL-cholesterol, mg/dl	37 123	1	51·9	0·3	51·7	0·4	51·5	0·4	51·0	0·3	51·4	0·4	–0·17	0·09	0·0592	–0·41	0·25	0·1013
		2	51·9	0·3	51·7	0·4	51·5	0·4	51·0	0·3	51·4	0·4	–0·17	0·09	0·0512	–0·42	0·25	0·0897
		3	51·9	0·3	51·7	0·4	51·5	0·4	51·0	0·3	51·4	0·4	–0·17	0·09	0·0494	–0·43	0·25	0·0867
		4	52·0	0·3	51·8	0·4	51·5	0·4	51·1	0·3	51·5	0·4	–0·17	0·08	0·0439	–0·40	0·25	0·1107
TAG, mg/dl^1^	16 913	1	130·6	2·8	128·3	3·3	129·0	2·9	130·9	3·5	129·4	3·0	–0·16	0·97	0·7152	0·32	2·60	0·9285
		2	130·9	2·8	128·4	3·3	129·1	2·9	130·8	3·5	128·9	3·0	–0·31	1·00	0·6644	–0·15	2·68	0·9800
		3	130·7	2·8	128·2	3·4	129·0	2·9	130·6	3·6	128·9	3·0	–0·29	1·00	0·6948	–0·06	2·69	0·9452
		4	130·7	2·8	128·2	3·4	129·0	2·9	130·6	3·6	128·9	3·0	–0·29	1·00	0·6799	–0·08	2·69	0·9890
Waist circumference, cm	37 761	1	97·0	0·3	97·0	0·4	97·9	0·3	98·7	0·4	98·0	0·5	0·36	0·09	**0·0001**	0·82	0·27	**0·0028**
		2	97·1	0·3	97·0	0·4	97·9	0·3	98·7	0·4	98·0	0·5	0·34	0·09	**0·0002**	0·77	0·28	**0·0065**
		3	97·2	0·3	97·1	0·4	98·0	0·3	98·8	0·4	98·0	0·5	0·32	0·09	**0·0003**	0·68	0·27	0·0123
		4	97·2	0·3	97·1	0·4	98·0	0·3	98·8	0·4	98·0	0·5	0·32	0·09	**0·0003**	0·68	0·27	0·0116

NHANES; National Health and Nutrition Examination Survey; cup eq, cup equivalent; HOMA-IR, Homeostatic Model Assessment for Insulin Resistance; BP, blood pressure.

Values are least squares means (se). Model 1 is adjusted for age, gender, ethnicity, physical activity, poverty:income ratio, smoking status and alcohol intake. Model 2 is further adjusted for energy intake. Model 3 is further adjusted for saturated fat and sodium intake. Model 4 is further adjusted for total carbohydrate intake. HOMA-IR = fasting insulin (µU/ml) × fasting glucose (mmol/l)/22·5. Significance was set at *P* < 0·01. ^1^Indicates variable was log-transformed to generate *P*-values; means, se and beta values are derived from the non-transformed model.

**Table 3.** Associations of average white potato intake with cardiometabolic risk factors in US adults with diabetes: NHANES 2001–2018 Table 3 long description.

			Quintiles of average white potato intake (cup eq)										Quintile trend			Linear trend		
	*n*	Model	1		2		3		4		5		*β*	se	*P*	*β*	se	*P*
			Mean	se	Mean	se	Mean	se	Mean	se	Mean	se						
Glucose, mg/dl^1^	2364	1	154·1	4·3	149·7	4·6	152·8	4·8	161·9	5·3	165·6	6·4	2·91	1·45	0·0233	11·35	5·20	0·0214
		2	154·6	4·4	150·0	4·6	152·5	4·9	161·4	5·2	164·2	6·4	2·50	1·46	0·0556	10·01	5·22	0·0482
		3	154·7	4·4	150·2	4·6	152·4	4·9	161·5	5·2	164·3	6·4	2·48	1·47	0·0593	9·96	5·21	0·0493
		4	154·7	4·4	150·2	4·6	152·5	4·9	161·5	5·2	164·3	6·4	2·48	1·46	0·0586	9·96	5·21	0·0495
Glycated Hb, %^1^	5250	1	7·39	0·09	7·33	0·10	7·47	0·11	7·46	0·11	7·50	0·11	0·03	0·02	0·0781	0·05	0·06	0·3043
		2	7·40	0·09	7·34	0·10	7·46	0·11	7·45	0·11	7·48	0·12	0·02	0·02	0·1583	0·02	0·06	0·5497
		3	7·40	0·09	7·35	0·10	7·46	0·11	7·44	0·11	7·47	0·11	0·02	0·02	0·2065	0·01	0·06	0·6525
		4	7·40	0·09	7·35	0·10	7·46	0·11	7·44	0·11	7·47	0·11	0·02	0·02	0·2091	0·01	0·06	0·6493
Insulin, μU/ml^1^	2297	1	20·4	1·8	19·5	2·1	27·7	4·8	23·3	2·4	24·0	3·6	1·14	0·64	0·0796	2·08	2·09	0·2182
		2	20·5	1·8	19·5	2·1	27·6	4·8	23·2	2·4	23·7	3·6	1·05	0·62	0·1421	1·74	2·08	0·3916
		3	20·6	1·8	19·6	2·1	27·6	4·8	23·3	2·4	23·7	3·6	1·04	0·63	0·1380	1·70	2·09	0·3870
		4	20·6	1·8	19·6	2·2	27·6	4·8	23·2	2·4	23·8	3·6	1·03	0·63	0·1394	1·72	2·09	0·3794
HOMA-IR^1^	2295	1	8·09	0·86	7·59	0·95	10·61	1·91	9·64	1·09	10·14	1·63	0·60	0·32	0·0224	1·33	1·00	0·0511
		2	8·16	0·84	7·63	0·95	10·56	1·91	9·58	1·08	9·94	1·63	0·54	0·31	0·0482	1·10	0·98	0·1154
		3	8·20	0·86	7·66	0·96	10·57	1·91	9·62	1·09	9·97	1·63	0·53	0·31	0·0484	1·09	0·99	0·1165
		4	8·21	0·86	7·65	0·96	10·55	1·92	9·58	1·09	9·98	1·63	0·53	0·31	0·0490	1·10	0·98	0·1130
Systolic BP, mmHg	5279	1	132·3	0·9	132·8	1·2	132·2	1·3	132·7	1·4	133·0	1·3	0·14	0·31	0·6582	0·26	0·87	0·7657
		2	132·2	0·9	132·8	1·2	132·3	1·3	132·8	1·4	133·3	1·4	0·22	0·32	0·4875	0·52	0·88	0·5567
		3	132·2	0·9	132·7	1·2	132·3	1·4	132·8	1·4	133·2	1·4	0·21	0·32	0·5010	0·49	0·89	0·5846
		4	132·2	0·9	132·7	1·2	132·3	1·4	132·8	1·4	133·2	1·4	0·22	0·32	0·4940	0·49	0·89	0·5847
Diastolic BP, mmHg	5236	1	68·8	0·6	68·9	0·8	69·1	0·7	69·8	0·7	68·1	0·8	–0·02	0·17	0·9310	–0·67	0·64	0·2997
		2	68·8	0·6	68·9	0·8	69·1	0·7	69·8	0·7	68·0	0·8	–0·05	0·17	0·7805	–0·81	0·67	0·2305
		3	68·8	0·6	68·9	0·8	69·1	0·7	69·8	0·7	68·0	0·8	–0·05	0·18	0·7785	–0·81	0·67	0·2289
		4	68·8	0·6	68·9	0·8	69·1	0·7	69·8	0·7	68·0	0·8	–0·05	0·18	0·7767	–0·81	0·67	0·2288
Total cholesterol, mg/dl	5157	1	188·1	2·1	189·8	3·3	189·8	2·5	188·1	3·2	189·8	4·0	0·26	0·88	0·7682	0·16	2·51	0·9504
		2	188·1	2·1	189·7	3·3	189·8	2·5	188·1	3·2	190·0	4·0	0·31	0·87	0·7230	0·27	2·54	0·9141
		3	188·1	2·1	189·7	3·3	189·7	2·5	188·0	3·2	190·1	3·9	0·32	0·84	0·7007	0·33	2·50	0·8961
		4	188·1	2·1	189·8	3·3	189·7	2·5	188·1	3·2	190·1	3·9	0·32	0·84	0·7055	0·33	2·50	0·8946
LDL-cholesterol, mg/dl	2226	1	103·1	2·4	103·0	3·2	105·4	2·8	105·7	3·3	107·8	3·7	1·11	0·99	0·2650	2·47	3·08	0·4248
		2	102·7	2·4	102·6	3·1	105·6	2·8	105·8	3·3	108·8	3·7	1·44	0·97	0·1424	3·62	3·08	0·2423
		3	102·8	2·3	103·1	3·1	105·2	2·9	105·5	3·3	109·0	3·7	1·37	0·94	0·1478	3·42	3·01	0·2575
		4	102·8	2·3	103·0	3·1	105·1	2·9	105·4	3·2	109·0	3·7	1·35	0·94	0·1510	3·42	3·00	0·2566
HDL-cholesterol, mg/dl	5157	1	47·4	0·6	47·7	0·8	48·2	1·0	48·0	1·0	48·0	1·0	0·17	0·21	0·4205	0·21	0·61	0·7320
		2	47·4	0·6	47·7	0·8	48·2	1·0	48·0	1·0	47·9	1·0	0·17	0·22	0·4505	0·17	0·63	0·7838
		3	47·4	0·6	47·6	0·8	48·2	1·0	48·0	1·0	48·0	1·0	0·18	0·22	0·4286	0·20	0·64	0·7576
		4	47·4	0·6	47·8	0·8	48·2	1·0	48·1	1·0	47·9	1·0	0·17	0·22	0·4488	0·21	0·64	0·7474
TAG, mg/dl^1^	2316	1	175·0	12·5	171·3	12·3	184·4	14·3	175·6	15·1	186·9	19·0	2·44	3·70	0·6202	27·55	20·03	0·1901
		2	176·2	12·7	172·1	12·5	183·6	13·9	174·5	14·5	183·6	18·2	1·38	3·65	0·7213	24·60	20·10	0·2146
		3	175·9	12·8	171·5	12·3	183·8	14·0	174·5	14·4	183·3	18·4	1·46	3·65	0·7073	24·78	20·02	0·2078
		4	176·0	12·8	171·3	12·3	183·5	14·1	173·9	14·2	183·6	18·4	1·41	3·70	0·7238	24·93	20·00	0·2043
Waist circumference, cm	5099	1	105·4	0·8	104·6	0·9	105·4	1·1	106·3	1·2	107·1	1·2	0·42	0·24	0·0880	1·80	0·70	0·0110
		2	105·7	0·8	104·8	0·9	105·4	1·1	106·1	1·2	106·6	1·2	0·23	0·24	0·3457	1·23	0·71	0·0837
		3	105·8	0·8	104·9	0·9	105·3	1·1	106·0	1·2	106·6	1·2	0·18	0·24	0·4411	1·13	0·70	0·1103
		4	105·8	0·8	105·0	0·9	105·4	1·1	106·1	1·2	106·6	1·2	0·18	0·24	0·4468	1·13	0·70	0·1074

NHANES; National Health and Nutrition Examination Survey; cup eq, cup equivalent; HOMA-IR, Homeostatic Model Assessment for Insulin Resistance; BP, blood pressure.

Values are least squares means (se). Model 1 is adjusted for age, gender, ethnicity, physical activity, poverty:income ratio, smoking status and alcohol intake. Model 2 is further adjusted for energy intake. Model 3 is further adjusted for saturated fat and sodium intake. Model 4 is further adjusted for total carbohydrate intake. HOMA-IR = fasting insulin (µU/ml) × fasting glucose (mmol/l)/22·5. Significance was set at *P* < 0·01. ^1^Indicates variable was log-transformed to generate *P*-values; means, SE, and beta values are derived from the non-transformed model.

Dietary patterns created by the cluster analysis using mean percent calories consumed for each food group are presented in Table 1. In the population without diabetes, glycated Hb was lower in those who primarily consumed baked/boiled potatoes with a higher percentage of calories from protein foods and a lower percentage from mixed dishes (cluster 1, *P* = 0·0008) compared with those with no white potato intake (cluster 0; Table 4). HDL-cholesterol was lower and insulin concentrations and waist circumference were higher in those who primarily consumed fried potatoes with a higher percentage of calories from mixed dishes and non-alcoholic beverages and lower percentage from dairy products, protein, whole and refined grains, snacks/sweets, fruits and vegetables (cluster 1, *P* < 0·0001 for all) compared with those with no white potato intake. Insulin and HOMA-IR were higher in those who primarily consumed mashed potatoes with a higher percentage of calories from protein foods and snacks/sweets with a lower percentage from mixed dishes (insulin: cluster 2, *P* < 0·0001; HOMA-IR: cluster 2, *P* = 0·0001) compared with those with no white potato intake. Waist circumference was higher in those who consumed mashed potatoes with a higher percentage of calories from mixed dishes and a lower percentage from dairy products, refined grains, fruit and non-alcoholic beverages (cluster 1, *P* = 0·0074) compared with those with no white potato intake. Waist circumference was higher in those who consumed chips with a higher percentage of calories from mixed dishes and a lower percentage from dairy products, protein foods, refined grains, snacks/sweets, fruits, vegetables and alcoholic beverages (cluster 2, *P* = 0·0007) compared with those with no white potato intake. For the baked/boiled clusters, waist circumference did not differ when compared with the no potato intake cluster. In the population with diagnosed diabetes, HDL-cholesterol was lower in those consuming baked/boiled potatoes with a higher percentage of calories from mixed dishes and lower percentage from dairy products, protein foods, refined grains, snacks/sweets, fats/oils and fruit (cluster 1, *P* = 0·0027) compared with those with no white potato intake, although this comparison should be interpreted with caution as the sample size for cluster 1 was very small (*n* 38, Table 5).

**Table 4.** Cardiometabolic risk factors by most consumed potato preparation method and dietary pattern cluster in US adults without diabetes: NHANES 2001–2018 Table 4 long description.

	Baked/Boiled							Chips							Fried							Mashed						
	C0		C1		C2		*P*	C0		C1		C2		*P*	C0		C1		C2		*P*	C0		C1		C2		*P*
	Mean	se	Mean	se	Mean	se		Mean	se	Mean	se	Mean	se		Mean	se	Mean	se	Mean	se		Mean	se	Mean	se	Mean	se	
*n*	24 166		988		469			24 166		2635		1371			24 166		3411		2577			24 166		1051		2437		
Glucose, mg/dl^1^	100·1	0·3	100·3	1·0	98·2	0·9	0·2223	100·0	0·3	100·3	0·6	101·4	1·2	0·4611	99·8	0·3	101·1	0·6	99·4	0·5	0·1212	100·0	0·3	101·2	0·9	100·5	0·6	0·3083
Glycated Hb, %^1^	5·41	0·01	5·35	0·02*	5·37	0·03	**0·0008**	5·41	0·01	5·40	0·02	5·44	0·03	0·3710	5·40	0·01	5·43	0·02	5·41	0·02	0·1634	5·41	0·01	5·39	0·02	5·41	0·02	0·4868
Insulin, µU/ml^1^	11·1	0·2	11·1	0·7	11·3	1·0	0·5929	11·1	0·2	10·7	0·3	12·4	0·6	0·0536	11·1	0·2	12·1	0·4*	11·1	0·4	**<0·0001**	11·1	0·2	11·4	0·4	13·2	0·5*	**<0·0001**
HOMA-IR^1^	2·86	0·12	2·88	0·23	2·82	0·30	0·4927	2·86	0·11	2·72	0·13	3·32	0·25	0·1651	2·86	0·11	3·12	0·14	2·83	0·14	0·1059	2·85	0·13	2·97	0·17	3·40	0·18*	**0·0001**
Systolic BP, mmHg	122·1	0·2	121·6	0·9	121·2	0·9	0·5344	122·0	0·2	122·1	0·5	122·2	0·7	0·9881	121·8	0·2	122·4	0·4	122·2	0·5	0·3127	122·2	0·2	122·3	0·8	123·0	0·6	0·3445
Diastolic BP, mmHg	71·4	0·2	71·4	0·5	72·7	0·8	0·2745	71·5	0·2	72·2	0·4	71·9	0·5	0·1174	71·5	0·2	71·4	0·4	71·8	0·3	0·5726	71·4	0·2	71·1	0·5	71·6	0·4	0·7146
Total cholesterol, mg/dl	197·0	0·5	198·8	1·5	197·1	2·9	0·5129	196·7	0·5	198·1	1·4	196·4	1·5	0·5543	196·4	0·5	195·0	1·1	196·6	1·3	0·4267	197·0	0·5	197·0	2·2	198·1	1·3	0·7307
LDL-cholesterol, mg/dl	116·4	0·5	117·3	2·1	115·8	3·1	0·8987	116·3	0·5	116·5	1·4	115·1	1·9	0·8125	116·0	0·5	114·9	1·1	115·3	1·7	0·6160	116·4	0·5	110·0	2·1	116·8	1·5	0·0152
HDL-cholesterol, mg/dl	54·1	0·2	55·1	0·8	51·6	1·0	0·0151	54·1	0·2	54·1	0·5	53·5	0·7	0·7265	53·9	0·2	52·0	0·4*	52·9	0·6	**<0·0001**	54·2	0·2	53·3	0·9	53·4	0·4	0·1924
TAG, mg/dl^1^	125·4	1·8	128·0	5·8	124·2	8·5	0·5955	125·2	1·8	125·6	3·3	128·4	5·0	0·6896	125·1	1·8	122·7	3·7	128·5	4·2	0·5155	125·3	1·8	133·6	6·5	137·2	6·5	0·0854
Waist circumference, cm	96·9	0·2	95·7	0·7	97·0	1·0	0·2437	96·9 (0·2		96·0	0·4	99·3	0·8*	**0·0007**	96·8	0·2	99·5	0·5*	97·9	0·5	**<0·0001**	96·9	0·2	99·1	0·8*	97·9	0·5	**0·0074**

NHANES; National Health and Nutrition Examination Survey; C0, cluster 0; C1, cluster 1; C2, cluster 2; HOMA-IR, Homeostatic Model Assessment for Insulin Resistance; BP, blood pressure.

Values are least squares means (se). Variables were adjusted for age, gender, ethnicity, physical activity, poverty:income ratio, smoking status, alcohol intake and energy intake. HOMA-IR = fasting insulin (µU/ml) × fasting glucose (mmol/l)/22·5. Significance was set at *P* < 0·01. *P < 0·01 compared with cluster 0. ^1^Indicates variable was log-transformed to generate *P*-values; means, SE, and beta values are derived from the non-transformed model. Bolded text represents a significant *P*-value (*P* < 0.01).

**Table 5.** Cardiometabolic risk factors by most consumed potato preparation method and dietary pattern cluster in US adults with diabetes: NHANES 2001–2018 Table 5 long description.

	Baked/Boiled							Chips							Fried							Mashed						
	C0		C1		C2		*P*	C0		C1		C2		*P*	C0		C1		C2		*P*	C0		C1		C2		*P*
	Mean	se	Mean	se	Mean	se		Mean	se	Mean	se	Mean	se		Mean	se	Mean	se	Mean	se		Mean	se	Mean	se	Mean	se	
*n*	3481		38		210			3481		255		247			3481		493		223			3481		353		167		
Glucose, mg/dl^1^	156·2	2·7	174·7	32·6	153·8	9·0	0·7957	156·3	2·7	151·6	5·8	152·1	8·9	0·8156	157·2	2·7	161·8	5·4	169·8	7·7	0·2560	156·3	2·7	165·9	6·5	166·0	10·0	0·1841
Glycated Hb, %^1^	7·29	0·11	6·75	0·48	7·31	0·21	0·4544	7·29	0·11	7·22	0·21	7·26	0·19	0·8601	7·30	0·11	7·35	0·14	7·39	0·19	0·6720	7·28	0·10	7·34	0·16	7·44	0·23	0·5851
Insulin, µU/ml^1^	19·4	0·9	21·6	4·0	14·3	2·8	0·1114	19·3	0·9	20·8	3·2	22·7	3·2	0·4231	19·4	0·9	24·2	3·2	23·4	3·4	0·5481	19·3	0·9	26·8	5·1	18·6	1·9	0·2476
HOMA-IR^1^	7·93	0·47	10·40	4·04	6·12	1·60	0·4649	7·89	0·46	7·77	1·21	9·27	1·84	0·5532	7·95	0·46	10·12	1·34	10·09	1·48	0·1145	7·86	0·45	11·40	2·03	7·83	0·97	0·2711
Systolic BP, mmHg	131·3	0·5	130·1	2·9	131·2	1·9	0·9262	131·1	0·5	130·9	2·0	130·2	1·4	0·8408	130·8	0·5	130·8	1·3	134·4	2·7	0·4039	131·2	0·5	131·2	1·5	130·0	2·4	0·8738
Diastolic BP, mmHg	69·6	0·3	69·2	1·8	68·2	0·9	0·3881	69·8	0·3	69·8	0·9	68·8	0·9	0·4959	69·9	0·3	70·2	0·8	70·1	1·3	0·9218	69·6	0·3	67·7	1·4	71·1	1·2	0·1988
Total cholesterol, mg/dl	185·8	1·4	189·5	13·0	184·6	4·6	0·9334	185·7	1·4	187·5	5·0	179·4	4·6	0·3639	186·2	1·4	184·3	3·6	188·4	4·7	0·7794	185·4	1·4	186·0	5·8	182·0	5·8	0·8479
LDL-cholesterol, mg/dl	100·6	1·8	115·6	11·5	104·5	6·5	0·4145	100·5	1·8	102·5	4·3	104·5	5·1	0·7123	100·6	1·8	99·6	2·8	105·1	4·9	0·6184	100·3	1·8	107·8	4·0	110·1	7·4	0·1718
HDL-cholesterol, mg/dl	47·0	0·4	40·8	1·7*	46·8	1·3	**0·0027**	46·9	0·4	50·0	1·7	49·1	1·5	0·0839	46·9	0·4	49·3	0·9	48·1	2·0	0·0733	46·9	0·4	47·3	0·9	46·3	1·4	0·7905
TAG, mg/dl^1^	175·2	7·3	154·6	26·8	157·0	14·8	0·8004	175·6	7·4	163·3	15·1	157·4	17·3	0·4534	178·1	8·0	175·6	17·4	182·7	25·5	0·8044	175·3	7·2	159·4	10·4	179·6	14·1	0·2115
Waist circumference, cm	110·1	0·5	113·8	2·4	108·7	2·1	0·2474	110·3	0·5	109·3	1·7	110·9	2·2	0·8179	110·4	0·5	109·7	1·0	113·6	2·4	0·3030	110·3	0·5	109·9	1·9	111·0	2·1	0·9131

NHANES; National Health and Nutrition Examination Survey; C0, cluster 0; C1, cluster 1; C2, cluster 2; HOMA-IR, Homeostatic Model Assessment for Insulin Resistance; BP, blood pressure.

Values are least squares means (se). Variables were adjusted for age, gender, ethnicity, physical activity, poverty:income ratio, smoking status, alcohol intake and energy intake. HOMA-IR = fasting insulin (µU/ml) × fasting glucose (mmol/l)/22·5. Significance was set at *P* < 0·01. *P < 0·01 compared with cluster 0. ^1^Indicates variable was log-transformed to generate *P*-values; means, SE, and beta values are derived from the non-transformed model. Bolded text represents a significant *P*-value (*P* < 0.01).

In individuals without diagnosed diabetes, the HEI total score was greater (*P* < 0·0001) in those who consumed baked and boiled potatoes within cluster 1 (online Supplementary Table 7), when compared with individuals who did not consume white potatoes. The HEI total score was lower (*P* < 0·0001) in individuals who consumed chips within cluster 2, when compared with individuals who did not consume white potatoes. The HEI total score was lower (*P* < 0·0001) in individuals who consumed fried potatoes within cluster 1, when compared with individuals who did not consume white potatoes. The HEI total score was lower (*P* < 0·0001) in individuals who consumed mashed potatoes within cluster 1, when compared with individuals who did not consume white potatoes. In individuals with diagnosed diabetes, there were no cluster differences in the total HEI score for any potato preparation method. In individuals without diagnosed diabetes, the HEI total vegetable score was higher in clusters that included baked and boiled white potato, chips, fried white potato and mashed white potato intake compared with clusters with no white potato intake. Similarly, in individuals with diagnosed diabetes, the HEI total vegetable score was higher in clusters that included baked and boiled white potato, chips (cluster 1 only), fried white potato (cluster 1 only) and mashed white potato intake compared with clusters with no white potato intake.

## Discussion

To our knowledge, this is the first cross-sectional study to investigate total white potato consumption and dietary patterns containing white potatoes prepared by various methods in relation to diet quality and cardiometabolic health in populations with and without diagnosed diabetes using a large nationally representative sample of US adults. We found that (1) white potatoes were most commonly consumed in fried form in those with and without diabetes; (2) greater white potato intake was associated with greater absolute energy, macronutrient, cholesterol, sodium, potassium and vegetable intake in those with and without diabetes; (3) in the population without diabetes, greater white potato intake was associated with a lower total HEI score and higher waist circumference, insulin, glucose and HOMA-IR (model 4); (4) in the population with diabetes, there were no associations between white potato intake, total HEI score and markers of cardiometabolic health; (5) dietary patterns with chips, fried potatoes or mashed potatoes, but not baked/boiled potatoes, were associated with higher waist circumference compared with no white potato intake in individuals without diagnosed diabetes; and (6) cluster analysis revealed variable relationships between dietary patterns containing white potatoes prepared by various methods and diet quality and markers of cardiometabolic health.

Our results indicate that most American adults consume potatoes in fried form with added total and saturated fats and sodium, which reduces nutritional value; this finding is consistent with other studies^(26–28)^. In a cross-sectional study using NHANES data to determine differences in dietary intake among adults with and without diabetes, white potatoes (cooking method not specified) were ranked as the 8th highest energy contributing food or beverage in individuals with diabetes taking insulin (*n* 774), while French fries were the 5th highest energy contributing food or beverage in those with T2D not using insulin (*n* 2758) and 3rd highest in those without diabetes (*n* 17 796)^(26)^. These findings indicate that fried potatoes are contributing a considerable amount of energy to the American diet, while white potatoes are infrequently consumed as a whole food with the skin, which contains many beneficial nutrients and bioactive compounds^(29)^. In line with this finding, we show that greater white potato intake is associated with greater total energy, carbohydrate, total sugar, fat, cholesterol and sodium intake in individuals with and without diabetes and worse diet quality in those without diabetes. Other cross-sectional studies report similar positive associations between white potato consumption and total energy, fat, saturated fat and sodium intake^(30–32)^. Greater total energy, fat (particularly saturated) and carbohydrate intake (particularly refined sources) have a well-established link to increased cardiometabolic risk^(33,34)^.

In the present study, in the population without diabetes, greater total white potato intake was associated with a lower total HEI score and higher waist circumference, insulin, glucose and HOMA-IR. Furthermore, an analysis of three prospective cohort studies showing that greater consumption of potatoes (baked, boiled or mashed potatoes and French fries) was associated with an increased risk of developing T2D (with the greatest association with French fries), independent of BMI and other risk factors^(35)^. Interestingly, in the present study, after adjustment for BMI, white potato intake was no longer associated with fasting glucose, insulin or HOMA-IR, suggesting that BMI may mediate the relationship between white potato intake and insulin resistance observed in our cross-sectional analysis. Our findings were also influenced by the way covariates were expressed (i.e. absolute *v*. energy-adjusted sodium, saturated fat and carbohydrate intake), with energy-adjusted nutrient intake variables negating the relationships between white potato intake and fasting glucose, insulin and HOMA-IR. These findings are more in line with a prospective cohort analysis showing that higher total potato intake (≥ 4 cup equivalents/week), including both non-fried and fried potato consumption, was not associated with risk of developing T2D, hypertension or hypertriglyceridaemia^(36)^. Investigating dietary patterns containing white potatoes prepared by different methods (e.g. cluster analysis) may provide an alternative strategy to understand the relationships between white potatoes and cardiometabolic health.

In individuals without diabetes, cluster analysis indicated that diet quality and total vegetable intake were higher and glycated Hb was lower in those who primarily consumed baked/boiled potatoes with a high percentage of calories from protein foods and a low percentage from mixed dishes, when compared with individuals consuming no potatoes. However, HDL-cholesterol was lower and insulin concentrations higher in those who primarily consumed fried potatoes with a high percentage of calories from mixed dishes (e.g. lasagne, quesadillas or casseroles) and non-alcoholic beverages and a low percentage from dairy products, protein, grains, snacks/sweets and produce. Moreover, insulin and HOMA-IR were higher in individuals who primarily consumed mashed potatoes with a high percent of calories from protein foods and snacks/sweets and low percent from mixed dishes compared to those with no white potato intake. In a study^(37)^ using NHANES data, greater potato consumption was associated with lower HDL cholesterol levels, but the authors did not distinguish between various preparation methods. In a prospective cohort study, boiled potato intake had no relationship with all-cause or CVD mortality in both men and women.^(38)^ Similarly in a longitudinal study^(39)^, participants who reported having the highest consumption of unfried potatoes (compared to the lowest group) did not show an increased risk of mortality, yet participants who consumed fried potatoes 2-3 times per week had an increased risk of mortality. Findings from our study highlight the importance of potato preparation methods, which are differentially associated with cardiometabolic outcomes, highlighting the need for further investigation into potato preparation methods and dietary patterns and their implications on metabolic health.

In individuals without diabetes, waist circumference was higher in those who primarily consumed chips with a higher percentage of calories from mixed dishes and lower percentage from dairy products, protein foods, refined grains, snacks/sweets, produce and alcoholic beverages; those who consumed primarily fried potatoes with a higher percentage of calories from mixed dishes and non-alcoholic beverages and lower percentage from dairy products, protein, grains, snacks/sweets and produce; and those who consumed primarily mashed potatoes with a higher percentage of calories from mixed dishes and lower percentage from dairy products, grains, fruit and non-alcoholic beverages compared with individuals who did not consume white potatoes. Epidemiological studies indicate that a higher intake of potatoes, particularly in the fried form, is linked to increased abdominal adiposity^(40,41)^. In cross-sectional studies of adolescent females, potato consumption (in all forms) greater than once a week was associated with a higher prevalence of abdominal adiposity and overweight or obesity^(31)^. Many studies have acknowledged the strong association between consuming French fries and weight gain, contributing to overweight or obesity^(39,42–43)^. However, we found no differences in waist circumference between clusters consuming baked/boiled potatoes and the cluster with no white potato consumption. Our results indicate the importance of dietary intake patterns, in addition to preparation methods, when examining the relationship between abdominal adiposity and white potato intake.

In the population with diagnosed diabetes, there were no associations between total white potato intake, total HEI score and markers of cardiometabolic health. However, in this population with diagnosed diabetes, HDL-cholesterol was lower in those consuming primarily baked/boiled potatoes with a higher percentage of calories from mixed dishes and a lower percentage from dairy products, protein foods, refined grains, snacks/sweets, fats/oils and fruit compared with those consuming no white potatoes. In a cross-sectional study by Latcu et al.^(44)^, individuals with T2D consumed dietary patterns with less potatoes, in addition to less fats and oils, sugar, and snacks, when compared to dietary patterns of individuals with prediabetes. In another cross-sectional analysis^(45)^, greater consumption of carbohydrates, specifically potatoes, was inversely associated with cardiometabolic risk factors including systolic blood pressure and LDL-cholesterol in individuals with T2D. Our cross-sectional analysis of individuals with diagnosed diabetes should be interpreted cautiously as diabetes status was self-reported and did not distinguish between T1D and T2D and included a relatively small sample size, particularly for the cluster analysis. In addition, in individuals with diagnosed diabetes, 81 % were on glucose-lowering medication, 72 % on BP-lowering medication and 51 % on lipid-lowering medication (online Supplementary Table 8), which may overshadow any relationships between diet and health in these individuals.

Limitations of the present study include the cross-sectional design, use of self-reported dietary intake data and quintile analysis that may have resulted in too many groups with only small differences in potato intake. Moreover, differences in clusters observed may have been driven by other dietary components including the consumption of mixed dishes. Fruit and vegetable consumption within the cluster analysis likely had less impact on clustering due to low energy content compared with foods with higher energy content. Strengths of the study include the overall large sample size, cluster analysis by preparation methods, multiple statistical models and inclusion of populations with and without diagnosed diabetes. Furthermore, associations between dietary intake of white potatoes, preparation methods, dietary patterns and cardiometabolic risk factors reported in the present study may guide the design of future clinical trials that examine the effects of baked white potato (with the skin) intake on health outcomes.

In US adults with and without diabetes, white potatoes are most commonly consumed in fried form, which likely contributes to the observed association between greater total white potato intake and greater intakes of total and saturated fat, carbohydrates and sodium. This also may help explain the relationship between higher white potato intake and lower dietary quality, in addition to greater waist circumference, insulin concentrations and insulin resistance in individuals without diabetes. Moreover, in individuals without diagnosed diabetes, a dietary pattern containing baked and boiled potatoes was associated with lower glycated Hb, while a dietary pattern with fried potatoes, chips or mashed potatoes was associated with greater waist circumference. Clinical intervention studies are needed to understand the effects of white potato intake on cardiometabolic health in populations with and without T2D.

## Supporting information

10.1017/S0007114525106089.sm001 Akhavan et al. supplementary material Akhavan et al. supplementary material

## Supplementary material

For supplementary material/s referred to in this article, please visit https://doi.org/10.1017/S0007114525106089.

## Table 1 Long description

The table presents dietary patterns based on the mean percent calories from white potatoes and fifteen main food groups, categorized into clusters. The clusters are defined by potato intake and preparation methods: baked/boiled, chips, fried, and mashed. Cluster 0 represents no white potato intake. Each preparation method is split into two clusters to maximize differences in each food group. The table includes data for US adults with and without diagnosed diabetes, showing the mean percent caloric intake by food group category. The columns include Cluster, n, Potato, Dairy products, Protein foods, Mixed dishes, Refined grains, Whole grains, Snack/sweets, Fats/oils, Fruit, Vegetable, Bev, and Bev alcoholic. The rows are categorized by diabetes status and potato preparation method, with specific values for each food group.

Navigate back to Table 1.

## Table 2 Long description

The table presents data on the associations of average white potato intake with various cardiometabolic risk factors in US adults without diabetes, based on NHANES 2001-2018. It includes quintiles of average white potato intake, with columns for different models and their respective means and standard errors. The table covers factors such as glucose levels, glycated hemoglobin, insulin levels, HOMA-IR, systolic and diastolic blood pressure, total cholesterol, LDL-cholesterol, HDL-cholesterol, TAG levels, and waist circumference. Trends and linear trends are also provided for each factor. The data is organized into five quintiles, each showing the mean and standard error for each factor. Notable trends include the inverse relationship between white potato intake and certain demographic factors, as well as positive associations with others.

Navigate back to Table 2.

## Table 3 Long description

The table presents data on the associations of average white potato intake with various cardiometabolic risk factors in US adults, both with and without diabetes. It includes measurements such as glucose levels, glycated hemoglobin, insulin levels, HOMA-IR, systolic and diastolic blood pressure, total cholesterol, LDL-cholesterol, HDL-cholesterol, TAG levels, and waist circumference. The table is divided into quintiles of average white potato intake, with mean values and standard errors provided for each quintile. Trends across quintiles and linear trends are also included. The data shows how different levels of white potato intake correlate with these health metrics, highlighting significant trends and associations.

Navigate back to Table 3.

## Table 4 Long description

The table presents data on cardiometabolic risk factors across different potato preparation methods (Baked/Boiled, Chips, Fried, Mashed) and dietary pattern clusters (C0, C1, C2) in US adults without diabetes. It includes measurements such as glucose levels, glycated hemoglobin, insulin levels, HOMA-IR, systolic and diastolic blood pressure, total cholesterol, LDL-cholesterol, HDL-cholesterol, TAG, and waist circumference. Each category is further divided into mean, standard error, and sample size (n) for each preparation method and cluster. Notable trends include variations in glycated hemoglobin, insulin levels, and HDL-cholesterol across different preparation methods and clusters.

Navigate back to Table 4.

## Table 5 Long description

The table presents data on cardiometabolic risk factors across different potato preparation methods (Baked/Boiled, Chips, Fried, Mashed) and dietary pattern clusters (C0, C1, C2) in US adults with diabetes. It includes measurements such as glucose levels, glycated hemoglobin, insulin, HOMA-IR, systolic and diastolic blood pressure, total cholesterol, LDL-cholesterol, HDL-cholesterol, TAG, and waist circumference. Each category is further divided into mean and standard error values for each preparation method and dietary pattern cluster. Notable trends include variations in glucose levels, cholesterol, and blood pressure across different preparation methods and dietary patterns.

Navigate back to Table 5.
