# Supplementary material for: Associations of white potato intake and preparation methods with cardiometabolic health measures in US adults categorised by diabetes status
Source: Br J Nutr. 2025 Dec 29;135(7):760–73. doi: 10.1017/S0007114525106089 (PMC13246594; doi:10.1017/S0007114525106089)
Supplement: Akhavan et al. supplementary material [file S0007114525106089sup001.docx]

**Supplemental Figure 1. CONSORT diagram.**

NHANES 2001-2018, 19+ y

(n=49,639)

Analysis sample (n=44,576)

Excluded (n=5,063)

♦  Unreliable dietary recall status (n=3,548)

♦  Pregnant or lactating (n=1,515)

“Doctor told you have diabetes?”

Excluded (n=950)

♦  Answered “Borderline” or “Don’t know” (n=950)

Answered “Yes”

Answered “No”

Diagnosed with diabetes (n=5,467)

No diabetes

(n=38,159)

**Supplemental Table 1.** USDA food codes for top 25 white potato-containing foods consumed by US adults categorized by diabetes status: NHANES 2001–2018.

|  | No diabetes (n=38,159) | | Diagnosed with diabetes (n=5,467) | |
| --- | --- | --- | --- | --- |
| Rank | Food code | Description | Food code | Description |
| 1 | 71401030 | Potato, french fries, fast food | 71401030 | Potato, french fries, fast food |
| 2 | 71201010 | White potato, chips | 71201010 | White potato, chips |
| 3 | 71403000 | White potato, home fries | 71403000 | White potato, home fries |
| 4 | 71201015 | White potato chips, regular cut | 71201015 | White potato chips, regular cut |
| 5 | 71501000 | Potato, mashed, NFS | 71601010 | Potato salad with egg |
| 6 | 71401000 | Potato, french fries, NS as to fresh or frozen | 71200100 | Potato chips, plain |
| 7 | 71501020 | White potato, from fresh, mashed, made with milk and fat | 71501000 | Potato, mashed, NFS |
| 8 | 71501010 | Potato, mashed, from fresh, made with milk | 71501010 | Potato, mashed, from fresh, made with milk |
| 9 | 71101000 | Potato, baked, peel not eaten | 71101000 | Potato, baked, peel not eaten |
| 10 | 71101110 | White potato, baked, peel eaten, fat not added in cooking | 71101110 | White potato, baked, peel eaten, fat not added in cooking |
| 11 | 71601010 | Potato salad with egg | 71508010 | Potato, baked, peel eaten, with sour cream |
| 12 | 71405000 | White potato, hash brown, NS as to from fresh, frozen, or dry mix | 71405020 | White potato, hash brown, from frozen |
| 13 | 71201020 | White potato chips, ruffled, rippled, or crinkle cut | 71405000 | White potato, hash brown, NS as to from fresh, frozen, or dry mix |
| 14 | 71405020 | White potato, hash brown, from frozen | 71403020 | Potato, home fries, NFS |
| 15 | 71508005 | Potato, baked, peel eaten, with butter | 71501020 | White potato, from fresh, mashed, made with milk and fat |
| 16 | 71200100 | Potato chips, plain | 71507005 | Potato, baked, peel not eaten, with butter |
| 17 | 71501040 | Potato, mashed, from dry mix, made with milk | 71103000 | Potato, boiled, from fresh, peel not eaten, NS as to fat |
| 18 | 71305010 | White potato, scalloped | 71501017 | Potato, mashed, from restaurant, with gravy |
| 19 | 71603010 | Potato salad | 71603010 | Potato salad |
| 20 | 71508010 | Potato, baked, peel eaten, with sour cream | 71501040 | Potato, mashed, from dry mix, made with milk |
| 21 | 71103010 | Potato, boiled, from fresh, peel not eaten, fat not added in cooking | 71508005 | Potato, baked, peel eaten, with butter |
| 22 | 71103000 | Potato, boiled, from fresh, peel not eaten, NS as to fat | 71201020 | White potato chips, ruffled, rippled, or crinkle cut |
| 23 | 71201250 | White potato, chips, restructured, baked | 75652010 | Vegetable beef soup, home recipe |
| 24 | 71301020 | White potato, cooked, with cheese | 71200200 | Potato chips, ruffled, plain |
| 25 | 71405010 | Potato, hash brown, from fresh | 71305010 | White potato, scalloped |

**Supplemental Table 2.** Demographics according to average white potato intake in US adults categorized by diabetes status: NHANES 2001–2018.

|  | Quintiles of average white potato intake (cup eq) | | | | | Quintile trend | | Linear trend | |
| --- | --- | --- | --- | --- | --- | --- | --- | --- | --- |
|  | 1 | 2 | 3 | 4 | 5 | β ± SE | P | β ± SE | P |
| No diabetes (n=38,159) |  |  |  |  |  |  |  |  |  |
| White potato intake, cup eq | 0.000 ± 0.000 | 0.140 ± 0.001 | 0.337 ± 0.001 | 0.604 ± 0.002 | 1.319 ± 0.010 |  |  |  |  |
| Female, % | 52.5 ± 0.6 | 61.3 ± 0.8 | 53.4 ± 0.8 | 47.8 ± 0.8 | 37.3 ± 0.9 | -3.30 ± 0.24 | **<0.0001** | -12.66 ± 0.67 | **<0.0001** |
| Age, y | 45.2 ± 0.3 | 47.2 ± 0.4 | 45.5 ± 0.3 | 45.8 ± 0.4 | 45.7 ± 0.4 | 0.10 ± 0.10 | 0.3058 | -0.10 ± 0.27 | 0.7132 |
| Ethnicity, % |  |  |  |  |  |  |  |  |  |
| Mexican American | 9.3 ± 0.7 | 8.9 ± 0.7 | 8.0 ± 0.7 | 7.2 ± 0.6 | 6.0 ± 0.5 | -0.79 ± 0.12 | **<0.0001** | -2.25 ± 0.32 | **<0.0001** |
| Other Hispanic | 5.9 ± 0.5 | 6.4 ± 0.5 | 4.8 ± 0.4 | 3.8 ± 0.4 | 3.8 ± 0.4 | -0.62 ± 0.09 | **<0.0001** | -1.48 ± 0.27 | **<0.0001** |
| Non-Hispanic White | 67.5 ± 1.1 | 65.7 ± 1.4 | 68.3 ± 1.3 | 71.2 ± 1.4 | 73.5 ± 1.2 | 1.46 ± 0.24 | **<0.0001** | 4.43 ± 0.62 | **<0.0001** |
| Non-Hispanic Black | 10.3 ± 0.6 | 9.8 ± 0.6 | 11.7 ± 0.8 | 12.1 ± 0.8 | 11.6 ± 0.8 | 0.46 ± 0.13 | **0.0007** | 0.83 ± 0.34 | 0.0143 |
| Other Race | 6.9 ± 0.4 | 9.2 ± 0.7 | 7.1 ± 0.5 | 5.8 ± 0.4 | 5.1 ± 0.4 | -0.51 ± 0.11 | **<0.0001** | -1.54 ± 0.34 | **<0.0001** |
| Poverty income ratio, % |  |  |  |  |  |  |  |  |  |
| < 1.35 | 21.5 ± 0.6 | 21.0 ± 0.8 | 21.2 ± 0.8 | 19.7 ± 0.9 | 21.1 ± 0.8 | -0.22 ± 0.20 | 0.2635 | -0.40 ± 0.50 | 0.4271 |
| 1.35 – 1.85 | 8.9 ± 0.4 | 9.0 ± 0.6 | 8.7 ± 0.5 | 9.6 ± 0.5 | 8.5 ± 0.5 | -0.01 ± 0.13 | 0.9210 | -0.36 ± 0.36 | 0.3191 |
| > 1.85 | 62.6 ± 0.9 | 62.9 ± 1.1 | 64.2 ± 1.0 | 65.0 ± 1.1 | 64.4 ± 1.1 | 0.60 ± 0.25 | 0.0162 | 1.65 ± 0.69 | 0.0179 |
| Current smoker, % | 19.8 ± 0.6 | 16.3 ± 0.8 | 20.3 ± 0.8 | 22.7 ± 0.9 | 26.7 ± 0.9 | 1.58 ± 0.21 | **<0.0001** | 5.87 ± 0.64 | **<0.0001** |
| Physical activity, % |  |  |  |  |  |  |  |  |  |
| Sedentary | 23.7 ± 0.6 | 24.3 ± 0.9 | 25.5 ± 0.9 | 23.9 ± 0.9 | 24.5 ± 0.9 | 0.20 ± 0.22 | 0.3572 | 0.55 ± 0.62 | 0.3780 |
| Moderate | 32.4 ± 0.6 | 36.7 ± 1.1 | 35.5 ± 1.0 | 34.5 ± 1.0 | 32.1 ± 1.0 | 0.10 ± 0.25 | 0.6838 | -1.43 ± 0.67 | 0.0341 |
| Vigorous | 43.9 ± 0.8 | 38.9 ± 1.2 | 39.0 ± 1.1 | 41.6 ± 1.0 | 43.3 ± 1.3 | -0.31 ± 0.28 | 0.2689 | 0.86 ± 0.85 | 0.3118 |
| Diagnosed with diabetes (n=5,467) |  |  |  |  |  |  |  |  |  |
| White potato intake, cup eq | 0.000 ± 0.000 | 0.133 ± 0.003 | 0.336 ± 0.003 | 0.593 ± 0.004 | 1.254 ± 0.023 |  |  |  |  |
| Female, % | 51.4 ± 1.5 | 56.5 ± 2.4 | 50.4 ± 2.3 | 46.6 ± 2.8 | 39.9 ± 2.1 | -2.70 ± 0.60 | **<0.0001** | -9.84 ± 1.64 | **<0.0001** |
| Age, y | 59.8 ± 0.5 | 61.5 ± 0.7 | 60.3 ± 0.6 | 59.3 ± 0.6 | 60.1 ± 0.7 | -0.06 ± 0.18 | 0.7395 | -0.25 ± 0.54 | 0.6441 |
| Ethnicity, % |  |  |  |  |  |  |  |  |  |
| Mexican American | 10.6 ± 1.2 | 12.6 ± 1.7 | 8.0 ± 1.3 | 7.4 ± 1.0 | 5.8 ± 0.9 | -1.32 ± 0.31 | **<0.0001** | -2.84 ± 0.84 | **0.0009** |
| Other Hispanic | 6.5 ± 0.8 | 6.7 ± 1.0 | 4.2 ± 0.8 | 4.7 ± 0.9 | 4.6 ± 0.8 | -0.56 ± 0.24 | 0.0207 | -1.54 ± 0.62 | 0.0146 |
| Non-Hispanic White | 55.7 ± 2.0 | 53.8 ± 2.8 | 66.1 ± 2.5 | 67.0 ± 2.6 | 70.2 ± 2.2 | 4.00 ± 0.67 | **<0.0001** | 10.87 ± 1.74 | **<0.0001** |
| Non-Hispanic Black | 17.6 ± 1.2 | 14.0 ± 1.4 | 15.2 ± 1.6 | 14.9 ± 1.6 | 13.8 ± 1.4 | -0.82 ± 0.40 | 0.0411 | -2.79 ± 1.06 | **0.0095** |
| Other Race | 9.6 ± 1.0 | 12.9 ± 1.7 | 6.4 ± 1.0 | 5.9 ± 1.2 | 5.6 ± 1.3 | -1.30 ± 0.35 | **0.0004** | -3.70 ± 1.00 | **0.0003** |
| Poverty income ratio, % |  |  |  |  |  |  |  |  |  |
| < 1.35 | 28.7 ± 1.4 | 26.3 ± 1.8 | 21.3 ± 2.0 | 24.2 ± 2.0 | 22.4 ± 1.6 | -1.62 ± 0.50 | **0.0016** | -3.40 ± 1.48 | 0.0232 |
| 1.35 – 1.85 | 12.5 ± 1.0 | 13.7 ± 1.6 | 11.4 ± 1.5 | 10.2 ± 1.3 | 8.4 ± 1.2 | -1.01 ± 0.37 | **0.0070** | -2.95 ± 1.07 | **0.0064** |
| > 1.85 | 50.2 ± 1.8 | 52.4 ± 2.5 | 60.2 ± 2.5 | 57.9 ± 2.5 | 60.8 ± 2.1 | 2.76 ± 0.62 | **<0.0001** | 6.26 ± 1.79 | **0.0006** |
| Current smoker, % | 18.8 ± 1.5 | 12.9 ± 1.5 | 16.8 ± 2.2 | 16.6 ± 2.1 | 16.7 ± 1.8 | -0.38 ± 0.50 | 0.4463 | -0.96 ± 1.45 | 0.5083 |
| Physical activity, % |  |  |  |  |  |  |  |  |  |
| Sedentary | 40.4 ± 1.8 | 45.0 ± 2.7 | 36.9 ± 2.5 | 38.9 ± 2.5 | 42.6 ± 2.6 | -0.04 ± 0.72 | 0.9568 | 0.72 ± 2.29 | 0.7557 |
| Moderate | 38.9 ± 2.2 | 38.4 ± 2.4 | 42.9 ± 2.9 | 39.4 ± 3.0 | 35.9 ± 2.2 | -0.36 ± 0.78 | 0.6448 | -1.11 ± 2.36 | 0.6381 |
| Vigorous | 20.7 ± 1.4 | 16.6 ± 2.3 | 20.2 ± 2.4 | 21.7 ± 2.5 | 21.5 ± 2.3 | 0.41 ± 0.64 | 0.5270 | 0.41 ± 1.83 | 0.8222 |

Values are means ± SE. Significance was set at P<0.01.

**Supplemental Table 3.** Nutrient intake according to average white potato intake in US adults categorized by diabetes status: NHANES 2001–2018.

|  | Quintiles of average white potato intake (cup eq) | | | | | Quintile trend | | Linear trend | |
| --- | --- | --- | --- | --- | --- | --- | --- | --- | --- |
|  | 1 | 2 | 3 | 4 | 5 | β ± SE | P | β ± SE | P |
| No diabetes (n=28,247) |  |  |  |  |  |  |  |  |  |
| White potato intake, cup eq | 0.000 ± 0.000 | 0.140 ± 0.001 | 0.337 ± 0.001 | 0.604 ± 0.002 | 1.319 ± 0.010 |  |  |  |  |
| Energy, kcal | 1947 ± 12 | 1968 ± 16 | 2058 ± 17 | 2147 ± 20 | 2439 ± 24 | 104.84 ± 4.94 | **<0.0001** | 361.06 ± 16.88 | **<0.0001** |
| Protein, g | 76.5 ± 0.6 | 74.7 ± 0.8 | 77.6 ± 0.9 | 80.0 ± 0.9 | 88.7 ± 1.0 | 2.47 ± 0.20 | **<0.0001** | 9.66 ± 0.61 | **<0.0001** |
| Carbohydrate, g | 242 ± 2 | 246 ± 2 | 253 ± 2 | 264 ± 2 | 297 ± 3 | 11.66 ± 0.72 | **<0.0001** | 40.38 ± 2.37 | **<0.0001** |
| Fiber, g | 15.5 ± 0.2 | 14.9 ± 0.2 | 14.8 ± 0.2 | 15.2 ± 0.2 | 17.2 ± 0.2 | 0.25 ± 0.06 | **<0.0001** | 1.53 ± 0.18 | **<0.0001** |
| Total sugars, g | 112.1 ± 1.2 | 113.3 ± 1.4 | 115.9 ± 1.5 | 121.1 ± 1.6 | 132.9 ± 2.1 | 4.45 ± 0.43 | **<0.0001** | 14.48 ± 1.46 | **<0.0001** |
| Added sugars, g | 74.5 ± 1.0 | 77.6 ± 1.4 | 80.8 ± 1.3 | 86.0 ± 1.4 | 95.6 ± 1.9 | 4.78 ± 0.37 | **<0.0001** | 13.98 ± 1.24 | **<0.0001** |
| Total fat, g | 70.9 ± 0.6 | 72.0 ± 0.8 | 77.1 ± 0.8 | 81.1 ± 0.9 | 95.5 ± 1.2 | 5.29 ± 0.23 | **<0.0001** | 17.73 ± 0.82 | **<0.0001** |
| PUFA, g | 15.6 ± 0.2 | 16.3 ± 0.2 | 17.3 ± 0.2 | 18.2 ± 0.3 | 21.2 ± 0.3 | 1.23 ± 0.06 | **<0.0001** | 4.03 ± 0.20 | **<0.0001** |
| MUFA, g | 25.3 ± 0.2 | 25.8 ± 0.3 | 27.8 ± 0.3 | 29.3 ± 0.3 | 34.6 ± 0.5 | 2.02 ± 0.09 | **<0.0001** | 6.65 ± 0.32 | **<0.0001** |
| SFA, g | 23.1 ± 0.2 | 23.2 ± 0.3 | 24.9 ± 0.3 | 26.1 ± 0.3 | 30.9 ± 0.4 | 1.65 ± 0.09 | **<0.0001** | 5.62 ± 0.30 | **<0.0001** |
| Cholesterol, mg | 279 ± 3 | 274 ± 4 | 289 ± 4 | 304 ± 4 | 347 ± 5 | 14.21 ± 1.10 | **<0.0001** | 52.38 ± 3.84 | **<0.0001** |
| Sodium, mg | 3109 ± 25 | 3145 ± 29 | 3283 ± 34 | 3385 ± 35 | 3812 ± 41 | 149.39 ± 7.87 | **<0.0001** | 524.43 ± 28.76 | **<0.0001** |
| Potassium, mg | 2333 ± 19 | 2314 ± 24 | 2469 ± 25 | 2620 ± 27 | 3190 ± 29 | 176.62 ± 6.86 | **<0.0001** | 672.96 ± 19.24 | **<0.0001** |
| Diagnosed with diabetes (n=4,183) |  |  |  |  |  |  |  |  |  |
| White potato intake, cup eq | 0.000 ± 0.000 | 0.133 ± 0.003 | 0.336 ± 0.003 | 0.593 ± 0.004 | 1.254 ± 0.023 |  |  |  |  |
| Energy, kcal | 1639 ± 36 | 1659 ± 43 | 1827 ± 46 | 1917 ± 46 | 2124 ± 52 | 115.93 ± 12.27 | **<0.0001** | 381.90 ± 37.08 | **<0.0001** |
| Protein, g | 69.4 ± 1.4 | 65.8 ± 2.0 | 73.7 ± 2.2 | 77.0 ± 2.3 | 84.0 ± 2.5 | 3.56 ± 0.54 | **<0.0001** | 12.40 ± 1.68 | **<0.0001** |
| Carbohydrate, g | 197 ± 5 | 206 ± 6 | 215 ± 6 | 226 ± 6 | 246 ± 6 | 11.52 ± 1.66 | **<0.0001** | 39.91 ± 4.87 | **<0.0001** |
| Fiber, g | 15.5 ± 0.4 | 15.1 ± 0.5 | 15.3 ± 0.6 | 16.3 ± 0.6 | 17.9 ± 0.6 | 0.50 ± 0.14 | **<0.0001** | 2.57 ± 0.43 | **<0.0001** |
| Total sugars, g | 79.4 ± 3.0 | 86.3 ± 3.3 | 88.2 ± 3.5 | 90.9 ± 3.8 | 94.1 ± 3.3 | 3.62 ± 0.96 | **<0.0001** | 10.75 ± 2.87 | **<0.0001** |
| Added sugars, g | 45.6 ± 2.6 | 52.0 ± 2.9 | 54.2 ± 3.0 | 55.9 ± 3.6 | 57.4 ± 3.1 | 3.01 ± 0.72 | **<0.0001** | 7.55 ± 2.31 | **<0.0001** |
| Total fat, g | 63.4 ± 1.7 | 62.6 ± 2.1 | 73.9 ± 2.5 | 78.0 ± 2.5 | 89.2 ± 2.9 | 6.23 ± 0.62 | **<0.0001** | 20.00 ± 1.87 | **<0.0001** |
| PUFA, g | 14.6 ± 0.4 | 14.3 ± 0.5 | 16.3 ± 0.6 | 17.7 ± 0.7 | 21.2 ± 0.8 | 1.50 ± 0.20 | **<0.0001** | 4.94 ± 0.61 | **<0.0001** |
| MUFA, g | 22.6 ± 0.7 | 22.3 ± 0.8 | 26.8 ± 0.9 | 28.3 ± 1.0 | 32.5 ± 1.0 | 2.41 ± 0.22 | **<0.0001** | 7.63 ± 0.68 | **<0.0001** |
| SFA, g | 20.0 ± 0.6 | 20.2 ± 0.7 | 24.1 ± 0.9 | 24.9 ± 0.9 | 27.4 ± 0.9 | 1.86 ± 0.20 | **<0.0001** | 5.87 ± 0.60 | **<0.0001** |
| Cholesterol, mg | 259 ± 10 | 245 ± 13 | 311 ± 15 | 303 ± 14 | 348 ± 17 | 21.71 ± 3.67 | **<0.0001** | 68.32 ±11.10 | **<0.0001** |
| Sodium, mg | 2826 ± 62 | 2839 ± 85 | 3199 ± 97 | 3331 ± 109 | 3599 ± 98 | 193.37 ± 23.50 | **<0.0001** | 618.71 ± 66.12 | **<0.0001** |
| Potassium, mg | 2170 ± 45 | 2179 ± 53 | 2377 ± 63 | 2640 ± 60 | 3112 ± 74 | 214.94 ± 17.30 | **<0.0001** | 772.71 ± 50.18 | **<0.0001** |

Values are least square means ± SE. Variables were adjusted for age, gender, ethnicity, physical activity, PIR ratio, smoking status, alcohol intake. Significance was set at <0.01. All variables were log-transformed to generate p-values; means, SE, and beta values are derived from the non-transformed model. Abbreviations: PUFA, polyunsaturated fatty acid; MUFA monounsaturated fatty acid; SFA, saturated fatty acid.

**Supplemental Table 4.** Nutrient intake adjusted for energy intake according to average white potato intake in US adults categorized by diabetes status: NHANES 2001–2018.

|  | Quintiles of average white potato intake (cup eq) | | | | | Quintile trend | | Linear trend | |
| --- | --- | --- | --- | --- | --- | --- | --- | --- | --- |
|  | 1 | 2 | 3 | 4 | 5 | β ± SE | P | β ± SE | P |
| No diabetes (n=28,247) |  |  |  |  |  |  |  |  |  |
| White potato intake, cup eq | 0.000 ± 0.000 | 0.140 ± 0.001 | 0.337 ± 0.001 | 0.604 ± 0.002 | 1.319 ± 0.010 |  |  |  |  |
| Protein, g/1000 kcal^1^ | 40.5 ± 0.2 | 38.8 ± 0.3 | 38.5 ± 0.3 | 38.1 ± 0.2 | 37.2 ± 0.3 | -0.78 ± 0.07 | **<0.0001** | -1.96 ± 0.18 | **<0.0001** |
| % kcal^1^ | 16.2 ± 0.1 | 15.5 ± 0.1 | 15.4 ± 0.1 | 15.2 ± 0.1 | 14.9 ± 0.1 | -0.31 ± 0.03 | **<0.0001** | -0.79 ± 0.07 | **<0.0001** |
| Carbohydrate, g/1000 kcal | 125.8 ± 0.5 | 126.7 ± 0.6 | 124.9 ± 0.5 | 124.7 ± 0.4 | 124.3 ± 0.6 | -0.42 ± 0.16 | **0.0083** | -1.18 ± 0.44 | **0.0081** |
| % kcal | 50.3 ± 0.2 | 50.7 ± 0.2 | 49.9 ± 0.2 | 49.9 ± 0.2 | 49.7 ± 0.2 | -0.17 ± 0.06 | **0.0083** | -0.47 ± 0.18 | **0.0081** |
| Fiber, g/1000 kcal | 8.3 ± 0.1 | 7.9 ± 0.1 | 7.5 ± 0.1 | 7.4 ± 0.1 | 7.5 ± 0.1 | -0.23 ± 0.02 | **<0.0001** | -0.45 ± 0.06 | **<0.0001** |
| Total sugars,  g/1000 kcal | 58.4 ± 0.4 | 58.4 ± 0.5 | 57.0 ± 0.5 | 56.7 ± 0.5 | 54.7 ± 0.6 | -0.82 ± 0.14 | **<0.0001** | -2.81 ± 0.38 | **<0.0001** |
| Added sugars,  g/1000 kcal | 37.8 ± 0.4 | 39.0 ± 0.6 | 38.7 ± 0.5 | 39.3 ± 0.5 | 38.2 ± 0.6 | 0.23 ± 0.13 | 0.0849 | -0.17 ± 0.37 | 0.6564 |
| Total fat, g/1000 kcal | 35.7 ± 0.2 | 35.9 ± 0.2 | 36.8 ± 0.2 | 37.2 ± 0.2 | 38.2 ± 0.2 | 0.62 ± 0.05 | **<0.0001** | 1.69 ± 0.16 | **<0.0001** |
| % kcal | 32.1 ± 0.1 | 32.3 ± 0.2 | 33.1 ± 0.2 | 33.5 ± 0.2 | 34.4 ± 0.2 | 0.55 ± 0.05 | **<0.0001** | 1.52 ± 0.14 | **<0.0001** |
| PUFA, g/1000  kcal | 7.9 ± 0.1 | 8.2 ± 0.1 | 8.3 ± 0.1 | 8.4 ± 0.1 | 8.6 ± 0.1 | 0.16 ± 0.02 | **<0.0001** | 0.41 ± 0.06 | **<0.0001** |
| MUFA, g/1000  kcal | 12.7 ± 0.1 | 12.8 ± 0.1 | 13.2 ± 0.1 | 13.4 ± 0.1 | 13.8 ± 0.1 | 0.28 ± 0.02 | **<0.0001** | 0.73 ± 0.06 | **<0.0001** |
| SFA, g/1000 kcal | 11.6 ± 0.1 | 11.5 ± 0.1 | 11.8 ± 0.1 | 11.9 ± 0.1 | 12.3 ± 0.1 | 0.17 ± 0.02 | **<0.0001** | 0.51 ± 0.07 | **<0.0001** |
| Cholesterol, mg/1000 kcal | 147.0 ± 1.5 | 140.6 ± 1.8 | 143.2 ± 1.7 | 143.6 ± 1.9 | 144.7 ± 2.0 | -0.64 ± 0.52 | 0.2184 | -0.34 ± 1.44 | 0.8135 |
| Sodium, mg/1000 kcal^1^ | 1642.6 ± 9.6 | 1642.8 ± 12.2 | 1634.5 ± 12.1 | 1612.2 ± 10.1 | 1598.8 ± 11.0 | -10.62 ± 2.74 | 0.3324 | -27.77 ± 7.99 | 0.1292 |
| Potassium, mg/1000 kcal | 1277.2 ± 7.2 | 1251.7 ± 12.4 | 1268.5 ± 9.7 | 1291.1 ± 8.9 | 1380.7 ± 9.5 | 19.17 ± 2.38 | **<0.0001** | 88.74 ± 6.50 | **<0.0001** |
| Diagnosed with diabetes (n=4,183) |  |  |  |  |  |  |  |  |  |
| White potato intake, cup eq | 0.000 ± 0.000 | 0.133 ± 0.003 | 0.336 ± 0.003 | 0.593 ± 0.004 | 1.254 ± 0.023 |  |  |  |  |
| Protein, g/1000 kcal^1^ | 43.7 ± 0.6 | 40.7 ± 0.6 | 40.9 ± 0.8 | 41.0 ± 0.8 | 40.5 ± 0.7 | -0.76 ± 0.18 | **0.0019** | -1.93 ± 0.50 | **0.0043** |
| % kcal^1^ | 17.5 ± 0.3 | 16.3 ± 0.3 | 16.3 ± 0.3 | 16.4 ± 0.3 | 16.2 ± 0.3 | -0.30 ± 0.07 | **0.0019** | -0.77 ± 0.20 | **0.0043** |
| Carbohydrate, g/1000 kcal | 121.8 ± 1.1 | 125.7 ± 1.8 | 120.2 ± 1.6 | 120.1 ± 1.7 | 118.8 ± 1.5 | -0.92 ± 0.34 | **0.0078** | -2.16 ± 0.86 | 0.0128 |
| % kcal | 48.7 ± 0.4 | 50.3 ± 0.7 | 48.1 ± 0.6 | 48.0 ± 0.7 | 47.5 ± 0.6 | -0.37 ± 0.14 | **0.0078** | -0.86 ± 0.34 | 0.0128 |
| Fiber, g/1000 kcal^1^ | 9.7 ± 0.2 | 9.5 ± 0.2 | 8.8 ± 0.3 | 8.9 ± 0.2 | 8.8 ± 0.2 | -0.24 ± 0.05 | **0.0035** | -0.41 ± 0.13 | 0.1866 |
| Total sugars,  g/1000 kcal | 49.5 ± 1.1 | 52.0 ± 1.4 | 49.1 ± 1.4 | 47.7 ± 1.5 | 44.6 ± 1.1 | -1.14 ± 0.32 | **0.0005** | -3.95 ± 0.83 | **<0.0001** |
| Added sugars,  g/1000 kcal^1^ | 27.6 ± 1.1 | 29.6 ± 1.2 | 29.0 ± 1.4 | 28.0 ± 1.4 | 26.0 ± 1.1 | -0.30 ± 0.27 | 0.6960 | -1.72 ± 0.77 | 0.4658 |
| Total fat, g/1000 kcal | 37.7 ± 0.4 | 36.9 ± 0.6 | 39.5 ± 0.5 | 39.7 ± 0.6 | 40.6 ± 0.6 | 0.79 ± 0.13 | **<0.0001** | 2.15 ± 0.34 | **<0.0001** |
| % kcal | 33.9 ± 0.4 | 33.2 ± 0.5 | 35.6 ± 0.5 | 35.8 ± 0.5 | 36.6 ± 0.5 | 0.71 ± 0.12 | **<0.0001** | 1.94 ± 0.30 | **<0.0001** |
| PUFA, g/1000  kcal | 8.8 ± 0.1 | 8.5 ± 0.2 | 8.9 ± 0.2 | 9.1 ± 0.2 | 9.6 ± 0.2 | 0.20 ± 0.06 | **0.0017** | 0.63 ± 0.17 | **0.0003** |
| MUFA, g/1000  kcal | 13.4 ± 0.2 | 13.2 ± 0.2 | 14.3 ± 0.2 | 14.4 ± 0.3 | 14.7 ± 0.2 | 0.37 ± 0.05 | **<0.0001** | 0.99 ± 0.13 | **<0.0001** |
| SFA, g/1000 kcal | 11.8 ± 0.2 | 11.8 ± 0.2 | 12.8 ± 0.3 | 12.6 ± 0.3 | 12.5 ± 0.2 | 0.21 ± 0.06 | **0.0003** | 0.49 ± 0.14 | **0.0006** |
| Cholesterol, mg/1000 kcal^1^ | 165.2 ± 4.7 | 152.1 ± 6.5 | 173.2 ± 6.9 | 163.6 ± 6.3 | 164.9 ± 6.0 | 0.55 ± 1.55 | 0.0375 | 0.63 ± 4.18 | 0.0989 |
| Sodium, mg/1000 kcal^1^ | 1796.6 ± 31.0 | 1777.2 ± 31.5 | 1768.4 ± 26.5 | 1783.2 ± 33.0 | 1740.2 ± 25.3 | -11.05 ± 7.74 | 0.7947 | -31.99 ± 19.82 | 0.4346 |
| Potassium, mg/1000 kcal^1^ | 1417.1 ± 27.4 | 1425.2 ± 27.5 | 1392.7 ± 23.6 | 1465.5 ± 25.6 | 1556.0 ± 26.9 | 27.95 ± 6.92 | **<0.0001** | 108.85 ± 17.06 | **<0.0001** |

Values are least square means ± SE. Variables were adjusted for age, gender, ethnicity, physical activity, PIR ratio, smoking status, alcohol intake. Significance was set at <0.01. ^1^Indicates variable was log-transformed to generate p-values; means, SE, and beta values are derived from the non-transformed model.

Abbreviations: PUFA, polyunsaturated fatty acid; MUFA monounsaturated fatty acid; SFA, saturated fatty acid.

**Supplemental Table 5.** HEI-2020 component and total scores according to average white potato intake in US adults categorized by diabetes status: NHANES 2001–2018.

|  | Quintiles of average white potato intake (cup eq) | | | | | Quintile trend | | Linear trend | |
| --- | --- | --- | --- | --- | --- | --- | --- | --- | --- |
|  | 1 | 2 | 3 | 4 | 5 | β ± SE | P | β ± SE | P |
| No diabetes (n=28,247) |  |  |  |  |  |  |  |  |  |
| White potato intake, cup eq | 0.000 ± 0.000 | 0.140 ± 0.001 | 0.337 ± 0.001 | 0.604 ± 0.002 | 1.319 ± 0.010 |  |  |  |  |
| Total vegetables | 2.70 ± 0.03 | 2.90 ± 0.04 | 3.09 ± 0.03 | 3.41 ± 0.03 | 4.02 ± 0.03 | 0.30 ± 0.01 | **<0.0001** | 0.94 ± 0.02 | **<0.0001** |
| Greens and beans | 2.03 ± 0.04 | 1.97 ± 0.05 | 1.75 ± 0.05 | 1.57 ± 0.05 | 1.46 ± 0.05 | -0.15 ± 0.01 | **<0.0001** | -0.39 ± 0.03 | **<0.0001** |
| Total fruit | 2.36 ± 0.03 | 2.30 ± 0.05 | 2.14 ± 0.04 | 2.08 ± 0.04 | 1.92 ± 0.05 | -0.11 ± 0.01 | **<0.0001** | -0.29 ± 0.03 | **<0.0001** |
| Whole fruit | 2.45 ± 0.03 | 2.39 ± 0.06 | 2.22 ± 0.05 | 2.16 ± 0.05 | 2.07 ± 0.06 | -0.10 ± 0.01 | **<0.0001** | -0.23 ± 0.04 | **<0.0001** |
| Whole grains | 2.36 ± 0.04 | 2.11 ± 0.07 | 1.96 ± 0.06 | 1.73 ± 0.07 | 1.53 ± 0.07 | -0.21 ± 0.02 | **<0.0001** | -0.55 ± 0.05 | **<0.0001** |
| Dairy | 4.78 ± 0.06 | 4.65 ± 0.07 | 4.53 ± 0.08 | 4.26 ± 0.08 | 4.03 ± 0.08 | -0.18 ± 0.02 | **<0.0001** | -0.54 ± 0.05 | **<0.0001** |
| Total protein foods | 4.36 ± 0.02 | 4.44 ± 0.03 | 4.46 ± 0.03 | 4.46 ± 0.02 | 4.42 ± 0.03 | 0.02 ± 0.01 | **0.0002** | 0.04 ± 0.02 | 0.0248 |
| Seafood and plant protein | 2.71 ± 0.04 | 2.76 ± 0.05 | 2.58 ± 0.06 | 2.37 ± 0.06 | 2.14 ± 0.06 | -0.14 ± 0.01 | **<0.0001** | -0.39 ± 0.04 | **<0.0001** |
| Fatty acid ratio | 4.93 ± 0.06 | 5.11 ± 0.08 | 5.14 ± 0.08 | 5.13 ± 0.09 | 5.16 ± 0.08 | 0.06 ± 0.02 | **0.0033** | 0.11 ± 0.06 | 0.0532 |
| Sodium | 4.77 ± 0.06 | 4.60 ± 0.08 | 4.73 ± 0.08 | 4.83 ± 0.07 | 4.92 ± 0.08 | 0.04 ± 0.02 | 0.0497 | 0.13 ± 0.05 | 0.0135 |
| Refined grain | 5.46 ± 0.06 | 5.41 ± 0.09 | 5.90 ± 0.09 | 6.37 ± 0.08 | 7.11 ± 0.07 | 0.38 ± 0.02 | **<0.0001** | 1.20 ± 0.05 | **<0.0001** |
| Saturated fat | 6.46 ± 0.06 | 6.51 ± 0.07 | 6.30 ± 0.08 | 6.17 ± 0.08 | 5.88 ± 0.09 | -0.13 ± 0.02 | **<0.0001** | -0.40 ± 0.06 | **<0.0001** |
| Added sugar | 6.22 ± 0.06 | 6.07 ± 0.09 | 6.00 ± 0.08 | 5.91 ± 0.08 | 6.06 ± 0.10 | -0.06 ± 0.02 | **0.0018** | -0.05 ± 0.06 | 0.3920 |
| HEI-2020 total score | 51.6 ± 0.2 | 51.2 ± 0.3 | 50.8 ± 0.3 | 50.5 ± 0.3 | 50.7 ± 0.3 | -0.27 ± 0.07 | **0.0003** | -0.42 ± 0.21 | 0.0437 |
| Diagnosed with diabetes (n=4,183) |  |  |  |  |  |  |  |  |  |
| White potato intake, cup eq | 0.000 ± 0.000 | 0.133 ± 0.003 | 0.336 ± 0.003 | 0.593 ± 0.004 | 1.254 ± 0.023 |  |  |  |  |
| Total vegetables | 2.95 ± 0.08 | 3.23 ± 0.09 | 3.42 ± 0.09 | 3.95 ± 0.09 | 4.32 ± 0.08 | 0.33 ± 0.02 | **<0.0001** | 1.01 ± 0.05 | **<0.0001** |
| Greens and beans | 2.25 ± 0.10 | 2.11 ± 0.14 | 1.84 ± 0.13 | 1.95 ± 0.14 | 1.73 ± 0.14 | -0.12 ± 0.03 | **0.0001** | -0.33 ± 0.09 | **0.0002** |
| Total fruit | 2.42 ± 0.09 | 2.61 ± 0.13 | 2.36 ± 0.12 | 2.18 ± 0.13 | 2.20 ± 0.12 | -0.07 ± 0.03 | 0.0214 | -0.19 ± 0.08 | 0.0141 |
| Whole fruit | 2.71 ± 0.10 | 2.92 ± 0.11 | 2.71 ± 0.13 | 2.66 ± 0.15 | 2.48 ± 0.14 | -0.06 ± 0.03 | 0.1026 | -0.13 ± 0.09 | 0.1626 |
| Whole grains | 3.37 ± 0.16 | 3.08 ± 0.26 | 2.81 ± 0.23 | 2.87 ± 0.24 | 2.54 ± 0.23 | -0.19 ± 0.05 | **0.0002** | -0.53 ± 0.14 | **0.0002** |
| Dairy | 4.55 ± 0.15 | 4.54 ± 0.21 | 4.76 ± 0.19 | 4.57 ± 0.21 | 3.92 ± 0.18 | -0.10 ± 0.05 | 0.0366 | -0.39 ± 0.14 | **0.0080** |
| Total protein foods | 4.55 ± 0.04 | 4.57 ± 0.06 | 4.65 ± 0.06 | 4.59 ± 0.06 | 4.77 ± 0.05 | 0.04 ± 0.01 | **0.0007** | 0.13 ± 0.03 | **0.0001** |
| Seafood and plant protein | 2.99 ± 0.09 | 3.14 ± 0.12 | 2.78 ± 0.14 | 2.75 ± 0.13 | 2.69 ± 0.13 | -0.09 ± 0.03 | **0.0044** | -0.23 ± 0.10 | 0.0196 |
| Fatty acid ratio | 5.54 ± 0.17 | 5.33 ± 0.21 | 5.40 ± 0.22 | 5.56 ± 0.20 | 6.15 ± 0.19 | 0.11 ± 0.05 | 0.0213 | 0.40 ± 0.15 | **0.0065** |
| Sodium | 3.73 ± 0.14 | 3.68 ± 0.20 | 3.58 ± 0.17 | 3.54 ± 0.22 | 3.63 ± 0.18 | -0.04 ± 0.05 | 0.4284 | 0.02 ± 0.12 | 0.8944 |
| Refined grain | 5.05 ± 0.18 | 5.21 ± 0.22 | 5.85 ± 0.20 | 5.92 ± 0.20 | 7.20 ± 0.18 | 0.47 ± 0.05 | **<0.0001** | 1.55 ± 0.12 | **<0.0001** |
| Saturated fat | 6.30 ± 0.17 | 6.33 ± 0.19 | 5.68 ± 0.22 | 5.81 ± 0.21 | 5.92 ± 0.21 | -0.13 ± 0.05 | 0.0102 | -0.29 ± 0.14 | 0.0349 |
| Added sugar | 7.59 ± 0.13 | 7.35 ± 0.17 | 7.36 ± 0.21 | 7.56 ± 0.19 | 7.78 ± 0.17 | 0.04 ± 0.04 | 0.3669 | 0.22 ± 0.12 | 0.0626 |
| HEI-2020 total score | 54.0 ± 0.5 | 54.1 ± 0.9 | 53.2 ± 0.9 | 53.9 ± 0.9 | 55.3 ± 0.8 | 0.20 ± 0.18 | 0.2660 | 1.25 ± 0.46 | **0.0078** |

Values are least squares means ± SE. Variables were adjusted for age, gender, ethnicity, physical activity, PIR ratio, smoking status, alcohol intake. Significance was set at P<0.01.

**Supplemental Table 6.** Associations of average white potato intake with cardiometabolic risk factors in US adults categorized by diabetes status: NHANES 2001–2018.

|  |  |  | Quintiles of average white potato intake (cup eq) | | | | | Quintile trend | | Linear trend | |
| --- | --- | --- | --- | --- | --- | --- | --- | --- | --- | --- | --- |
|  | n | Model | 1 | 2 | 3 | 4 | 5 | β ± SE | P | β ± SE | P |
| No diabetes |  |  |  |  |  |  |  |  |  |  |  |
| Glucose, mg/dL^1^ | 17071 | 5 | 102.6 ± 0.3 | 102.5 ± 0.4 | 102.8 ± 0.5 | 102.4 ± 0.5 | 103.6 ± 0.6 | 0.15 ± 0.11 | 0.2011 | 0.82 ± 0.38 | 0.0259 |
|  |  | 6 | 102.5 ± 0.4 | 102.3 ± 0.4 | 103.2 ± 0.6 | 102.6 ± 0.5 | 103.7 ± 0.6 | 0.22 ± 0.12 | 0.0974 | 0.94 ± 0.39 | 0.0217 |
|  |  | 7 | 102.5 ± 0.4 | 102.3 ± 0.4 | 103.2 ± 0.6 | 102.6 ± 0.5 | 103.7 ± 0.6 | 0.22 ± 0.12 | 0.0965 | 0.94 ± 0.39 | 0.0214 |
|  |  | 8 | 102.6 ± 0.3 | 102.6 ± 0.4 | 102.8 ± 0.5 | 102.4 ± 0.5 | 103.6 ± 0.6 | 0.14 ± 0.12 | 0.2908 | 0.80 ± 0.38 | 0.0468 |
| Glycated hemoglobin, %^1^ | 37491 | 5 | 5.57 ± 0.01 | 5.56 ± 0.01 | 5.56 ± 0.01 | 5.56 ± 0.01 | 5.58 ± 0.02 | 0.00 ± 0.00 | 0.7962 | 0.00 ± 0.01 | 0.8398 |
|  |  | 6 | 5.6 ± 0.0 | 5.6 ± 0.0 | 5.6 ± 0.0 | 5.6 ± 0.0 | 5.6 ± 0.0 | 0.00 ± 0.00 | 0.3086 | 0.01 ± 0.01 | 0.4700 |
|  |  | 7 | 5.6 ± 0.0 | 5.6 ± 0.0 | 5.6 ± 0.0 | 5.6 ± 0.0 | 5.6 ± 0.0 | 0.00 ± 0.00 | 0.3163 | 0.01 ± 0.01 | 0.4827 |
|  |  | 8 | 5.6 ± 0.0 | 5.6 ± 0.0 | 5.6 ± 0.0 | 5.6 ± 0.0 | 5.6 ± 0.0 | 0.00 ± 0.00 | 0.8764 | 0.00 ± 0.01 | 0.8774 |
| Insulin, μU/mL^1^ | 16748 | 5 | 11.9 ± 0.2 | 11.7 ± 0.2 | 12.6 ± 0.3 | 12.3 ± 0.3 | 12.1 ± 0.3 | 0.10 ± 0.06 | 0.0412 | 0.16 ± 0.15 | 0.1758 |
|  |  | 6 | 11.6 ± 0.2 | 11.4 ± 0.3 | 12.8 ± 0.4 | 12.5 ± 0.4 | 12.1 ± 0.3 | 0.21 ± 0.07 | 0.0625 | 0.36 ± 0.20 | 0.1019 |
|  |  | 7 | 11.7 ± 0.2 | 11.4 ± 0.3 | 12.8 ± 0.4 | 12.5 ± 0.4 | 12.1 ± 0.3 | 0.20 ± 0.07 | 0.0627 | 0.36 ± 0.20 | 0.1030 |
|  |  | 8 | 11.9 ± 0.2 | 11.7 ± 0.2 | 12.6 ± 0.3 | 12.3 ± 0.3 | 12.1 ± 0.3 | 0.10 ± 0.06 | 0.2708 | 0.16 ± 0.15 | 0.2544 |
| HOMA-IR^1^ | 16748 | 5 | 3.13 ± 0.06 | 3.08 ± 0.06 | 3.32 ± 0.10 | 3.21 ± 0.08 | 3.21 ± 0.08 | 0.03 ± 0.02 | 0.0363 | 0.07 ± 0.05 | 0.0949 |
|  |  | 6 | 3.1 ± 0.1 | 3.0 ± 0.1 | 3.4 ± 0.1 | 3.3 ± 0.1 | 3.2 ± 0.1 | 0.06 ± 0.02 | 0.0586 | 0.12 ± 0.06 | 0.0679 |
|  |  | 7 | 3.1 ± 0.1 | 3.0 ± 0.1 | 3.4 ± 0.1 | 3.3 ± 0.1 | 3.2 ± 0.1 | 0.06 ± 0.02 | 0.0587 | 0.12 ± 0.06 | 0.0685 |
|  |  | 8 | 3.1 ± 0.1 | 3.1 ± 0.1 | 3.3 ± 0.1 | 3.2 ± 0.1 | 3.2 ± 0.1 | 0.03 ± 0.02 | 0.2519 | 0.07 ± 0.05 | 0.1552 |
| Systolic BP, mmHg | 37885 | 5 | 124.3 ± 0.3 | 124.0 ± 0.4 | 124.3 ± 0.4 | 124.7 ± 0.4 | 124.7 ± 0.4 | 0.11 ± 0.09 | 0.2448 | 0.45 ± 0.30 | 0.1296 |
|  |  | 6 | 124.4 ± 0.3 | 124.0 ± 0.5 | 124.5 ± 0.4 | 125.0 ± 0.4 | 124.9 ± 0.4 | 0.16 ± 0.09 | 0.0761 | 0.56 ± 0.29 | 0.0585 |
|  |  | 7 | 124.4 ± 0.3 | 124.0 ± 0.5 | 124.5 ± 0.4 | 125.0 ± 0.4 | 124.9 ± 0.4 | 0.16 ± 0.09 | 0.0760 | 0.56 ± 0.29 | 0.0584 |
|  |  | 8 | 124.3 ± 0.3 | 124.0 ± 0.4 | 124.3 ± 0.4 | 124.7 ± 0.4 | 124.7 ± 0.4 | 0.11 ± 0.09 | 0.2338 | 0.45 ± 0.30 | 0.1273 |
| Diastolic BP, mmHg | 37741 | 5 | 71.5 ± 0.2 | 70.8 ± 0.3 | 71.3 ± 0.3 | 71.9 ± 0.3 | 71.7 ± 0.3 | 0.07 ± 0.06 | 0.2662 | 0.28 ± 0.19 | 0.1345 |
|  |  | 6 | 71.5 ± 0.2 | 70.7 ± 0.3 | 71.3 ± 0.3 | 72.0 ± 0.3 | 71.7 ± 0.3 | 0.10 ± 0.06 | 0.1109 | 0.34 ± 0.19 | 0.0709 |
|  |  | 7 | 71.5 ± 0.2 | 70.7 ± 0.3 | 71.3 ± 0.3 | 72.0 ± 0.3 | 71.7 ± 0.3 | 0.10 ± 0.06 | 0.1083 | 0.35 ± 0.19 | 0.0688 |
|  |  | 8 | 71.5 ± 0.2 | 70.8 ± 0.3 | 71.3 ± 0.3 | 71.9 ± 0.3 | 71.7 ± 0.3 | 0.07 ± 0.06 | 0.2544 | 0.29 ± 0.19 | 0.1285 |
| Total cholesterol, mg/dL | 37124 | 5 | 197.6 ± 0.8 | 196.8 ± 1.0 | 196.7 ± 1.1 | 197.7 ± 1.0 | 197.0 ± 1.1 | -0.09 ± 0.22 | 0.6718 | 0.07 ± 0.78 | 0.9280 |
|  |  | 6 | 197.5 ± 0.8 | 196.6 ± 1.0 | 196.6 ± 1.1 | 197.7 ± 1.0 | 197.1 ± 1.1 | -0.03 ± 0.22 | 0.8948 | 0.24 ± 0.79 | 0.7574 |
|  |  | 7 | 197.5 ± 0.8 | 196.7 ± 1.0 | 196.6 ± 1.1 | 197.8 ± 1.0 | 197.2 ± 1.1 | -0.02 ± 0.22 | 0.9165 | 0.27 ± 0.79 | 0.7325 |
|  |  | 8 | 197.7 ± 0.8 | 196.8 ± 1.0 | 196.7 ± 1.1 | 197.7 ± 1.0 | 197.1 ± 1.1 | -0.10 ± 0.22 | 0.6505 | 0.04 ± 0.79 | 0.9582 |
| LDL cholesterol, mg/dL | 16635 | 5 | 119.2 ± 0.8 | 116.3 ± 1.1 | 117.4 ± 1.2 | 117.6 ± 1.2 | 116.0 ± 1.2 | -0.65 ± 0.27 | 0.0161 | -1.70 ± 0.82 | 0.0387 |
|  |  | 6 | 119.0 ± 0.8 | 116.0 ± 1.1 | 117.4 ± 1.2 | 117.6 ± 1.2 | 116.0 ± 1.3 | -0.60 ± 0.27 | 0.0274 | -1.56 ± 0.83 | 0.0606 |
|  |  | 7 | 119.0 ± 0.8 | 116.0 ± 1.1 | 117.4 ± 1.2 | 117.6 ± 1.2 | 116.0 ± 1.3 | -0.60 ± 0.27 | 0.0276 | -1.56 ± 0.83 | 0.0614 |
|  |  | 8 | 119.2 ± 0.8 | 116.4 ± 1.1 | 117.4 ± 1.2 | 117.6 ± 1.2 | 115.9 ± 1.2 | -0.68 ± 0.27 | 0.0124 | -1.77 ± 0.82 | 0.0316 |
| HDL cholesterol, mg/dL | 37123 | 5 | 51.9 ± 0.2 | 51.6 ± 0.4 | 51.7 ± 0.4 | 51.4 ± 0.3 | 51.7 ± 0.3 | -0.08 ± 0.08 | 0.3325 | -0.18 ± 0.24 | 0.4565 |
|  |  | 6 | 51.9 ± 0.3 | 51.7 ± 0.4 | 51.5 ± 0.4 | 51.1 ± 0.3 | 51.4 ± 0.4 | -0.17 ± 0.09 | 0.0506 | -0.43 ± 0.25 | 0.0879 |
|  |  | 7 | 52.0 ± 0.3 | 51.8 ± 0.4 | 51.6 ± 0.4 | 51.1 ± 0.3 | 51.6 ± 0.4 | -0.16 ± 0.08 | 0.0533 | -0.39 ± 0.25 | 0.1146 |
|  |  | 8 | 51.9 ± 0.2 | 51.6 ± 0.4 | 51.7 ± 0.4 | 51.5 ± 0.3 | 51.8 ± 0.3 | -0.08 ± 0.08 | 0.3570 | -0.18 ± 0.24 | 0.4554 |
| Triglycerides, mg/dL^1^ | 16913 | 5 | 131.6 ± 2.8 | 129.4 ± 3.3 | 128.6 ± 2.9 | 129.9 ± 3.7 | 128.4 ± 3.1 | -0.75 ± 1.00 | 0.2712 | -1.01 ± 2.68 | 0.6227 |
|  |  | 6 | 130.5 ± 2.8 | 128.0 ± 3.4 | 128.9 ± 2.9 | 130.7 ± 3.6 | 129.0 ± 3.0 | -0.22 ± 1.01 | 0.3036 | 0.03 ± 2.71 | 0.7607 |
|  |  | 7 | 130.5 ± 2.8 | 128.0 ± 3.4 | 128.9 ± 2.9 | 130.7 ± 3.6 | 129.0 ± 3.0 | -0.22 ± 1.01 | 0.3081 | 0.03 ± 2.70 | 0.7611 |
|  |  | 8 | 131.4 ± 2.8 | 129.2 ± 3.3 | 128.5 ± 2.9 | 130.0 ± 3.6 | 128.4 ± 3.1 | -0.67 ± 1.01 | 0.1253 | -0.90 ± 2.69 | 0.4723 |
| Waist circumference, cm | 37761 | 6 | 97.2 ± 0.3 | 97.1 ± 0.4 | 98.0 ± 0.3 | 98.8 ± 0.4 | 98.0 ± 0.5 | 0.31 ± 0.09 | **0.0005** | 0.66 ± 0.27 | 0.0150 |
|  |  | 7 | 97.2 ± 0.3 | 97.2 ± 0.4 | 98.0 ± 0.3 | 98.8 ± 0.4 | 98.0 ± 0.5 | 0.31 ± 0.09 | **0.0005** | 0.66 ± 0.27 | 0.0143 |
| Diagnosed with diabetes |  |  |  |  |  |  |  |  |  |  |  |
| Glucose, mg/dL^1^ | 2364 | 5 | 155.1 ± 4.4 | 149.7 ± 4.8 | 153.3 ± 5.0 | 161.3 ± 5.4 | 165.4 ± 6.5 | 2.56 ± 1.45 | 0.0505 | 10.48 ± 5.24 | 0.0404 |
|  |  | 6 | 154.5 ± 4.4 | 150.0 ± 4.6 | 152.7 ± 4.9 | 161.6 ± 5.2 | 164.3 ± 6.3 | 2.57 ± 1.48 | 0.2024 | 10.11 ± 5.21 | 0.0530 |
|  |  | 7 | 154.5 ± 4.4 | 150.0 ± 4.6 | 152.7 ± 4.9 | 161.6 ± 5.1 | 164.3 ± 6.4 | 2.57 ± 1.48 | 0.2114 | 10.11 ± 5.21 | 0.0564 |
|  |  | 8 | 154.9 ± 4.4 | 149.5 ± 4.7 | 153.5 ± 5.0 | 161.4 ± 5.3 | 165.4 ± 6.5 | 2.65 ± 1.46 | 0.1675 | 10.65 ± 5.24 | 0.0372 |
| Glycated hemoglobin, %^1^ | 5250 | 5 | 7.41 ± 0.09 | 7.35 ± 0.11 | 7.47 ± 0.11 | 7.45 ± 0.11 | 7.49 ± 0.11 | 0.02 ± 0.02 | 0.1664 | 0.02 ± 0.06 | 0.5691 |
|  |  | 6 | 7.4 ± 0.1 | 7.3 ± 0.1 | 7.5 ± 0.1 | 7.4 ± 0.1 | 7.5 ± 0.1 | 0.02 ± 0.02 | 0.1673 | 0.02 ± 0.06 | 0.5590 |
|  |  | 7 | 7.4 ± 0.1 | 7.3 ± 0.1 | 7.5 ± 0.1 | 7.4 ± 0.1 | 7.5 ± 0.1 | 0.02 ± 0.02 | 0.1623 | 0.02 ± 0.06 | 0.5534 |
|  |  | 8 | 7.4 ± 0.1 | 7.3 ± 0.1 | 7.5 ± 0.1 | 7.4 ± 0.1 | 7.5 ± 0.1 | 0.02 ± 0.02 | 0.1268 | 0.03 ± 0.06 | 0.4752 |
| Insulin, μU/mL^1^ | 2297 | 5 | 22.7 ± 1.9 | 21.5 ± 2.3 | 29.4 ± 5.0 | 24.2 ± 2.4 | 25.0 ± 3.4 | 0.78 ± 0.57 | 0.3503 | 1.23 ± 1.91 | 0.6187 |
|  |  | 6 | 20.5 ± 1.8 | 19.6 ± 2.2 | 27.6 ± 4.8 | 23.2 ± 2.4 | 23.7 ± 3.6 | 1.05 ± 0.62 | 0.6176 | 1.74 ± 2.09 | 0.7721 |
|  |  | 7 | 20.5 ± 1.8 | 19.6 ± 2.3 | 27.6 ± 4.8 | 23.2 ± 2.4 | 23.7 ± 3.6 | 1.04 ± 0.63 | 0.6291 | 1.74 ± 2.09 | 0.7734 |
|  |  | 8 | 22.5 ± 1.9 | 21.3 ± 2.4 | 29.4 ± 5.0 | 24.1 ± 2.4 | 24.9 ± 3.4 | 0.79 ± 0.57 | 0.8494 | 1.24 ± 1.90 | 0.9822 |
| HOMA-IR^1^ | 2295 | 5 | 9.10 ± 0.90 | 8.43 ± 1.01 | 11.34 ± 1.97 | 9.97 ± 1.13 | 10.56 ± 1.57 | 0.42 ± 0.29 | 0.1028 | 0.91 ± 0.92 | 0.1671 |
|  |  | 6 | 8.2 ± 0.9 | 7.7 ± 1.0 | 10.6 ± 1.9 | 9.6 ± 1.1 | 10.0 ± 1.6 | 0.54 ± 0.31 | 0.3582 | 1.11 ± 0.99 | 0.3227 |
|  |  | 7 | 8.1 ± 0.9 | 7.6 ± 1.0 | 10.5 ± 1.9 | 9.6 ± 1.1 | 9.9 ± 1.6 | 0.54 ± 0.31 | 0.3714 | 1.11 ± 0.99 | 0.3255 |
|  |  | 8 | 9.0 ± 0.9 | 8.3 ± 1.0 | 11.3 ± 2.0 | 9.9 ± 1.1 | 10.5 ± 1.6 | 0.44 ± 0.29 | 0.4583 | 0.92 ± 0.92 | 0.3959 |
| Systolic BP, mmHg | 5279 | 5 | 132.2 ± 0.9 | 132.9 ± 1.2 | 132.5 ± 1.4 | 132.8 ± 1.4 | 133.2 ± 1.4 | 0.20 ± 0.32 | 0.5180 | 0.33 ± 0.87 | 0.7047 |
|  |  | 6 | 132.2 ± 0.9 | 132.7 ± 1.2 | 132.3 ± 1.4 | 132.8 ± 1.4 | 133.2 ± 1.4 | 0.24 ± 0.32 | 0.4551 | 0.54 ± 0.89 | 0.5428 |
|  |  | 7 | 132.1 ± 0.9 | 132.6 ± 1.2 | 132.3 ± 1.4 | 132.8 ± 1.4 | 133.3 ± 1.4 | 0.24 ± 0.32 | 0.4476 | 0.55 ± 0.89 | 0.5345 |
|  |  | 8 | 132.2 ± 0.9 | 132.8 ± 1.2 | 132.5 ± 1.4 | 132.8 ± 1.4 | 133.3 ± 1.3 | 0.23 ± 0.31 | 0.4722 | 0.39 ± 0.87 | 0.6524 |
| Diastolic BP, mmHg | 5236 | 5 | 68.9 ± 0.6 | 68.9 ± 0.8 | 69.2 ± 0.7 | 69.9 ± 0.7 | 68.0 ± 0.8 | -0.05 ± 0.18 | 0.7783 | -0.82 ± 0.68 | 0.2301 |
|  |  | 6 | 68.8 ± 0.6 | 68.9 ± 0.8 | 69.1 ± 0.7 | 69.8 ± 0.7 | 68.0 ± 0.8 | -0.05 ± 0.17 | 0.7706 | -0.81 ± 0.67 | 0.2303 |
|  |  | 7 | 68.8 ± 0.6 | 68.9 ± 0.8 | 69.0 ± 0.7 | 69.8 ± 0.7 | 68.0 ± 0.8 | -0.05 ± 0.17 | 0.7859 | -0.80 ± 0.68 | 0.2359 |
|  |  | 8 | 68.9 ± 0.6 | 68.9 ± 0.8 | 69.2 ± 0.7 | 69.9 ± 0.7 | 68.0 ± 0.8 | -0.05 ± 0.18 | 0.7794 | -0.82 ± 0.69 | 0.2333 |
| Total cholesterol, mg/dL | 5157 | 5 | 187.4 ± 2.1 | 189.2 ± 3.4 | 189.4 ± 2.5 | 187.9 ± 3.2 | 189.6 ± 3.9 | 0.41 ± 0.84 | 0.6279 | 0.49 ± 2.49 | 0.8428 |
|  |  | 6 | 188.3 ± 2.1 | 189.9 ± 3.3 | 189.6 ± 2.5 | 188.1 ± 3.2 | 189.9 ± 4.0 | 0.24 ± 0.84 | 0.7742 | 0.13 ± 2.50 | 0.9571 |
|  |  | 7 | 188.3 ± 2.1 | 190.1 ± 3.3 | 189.7 ± 2.5 | 188.2 ± 3.2 | 189.9 ± 4.0 | 0.24 ± 0.84 | 0.7789 | 0.12 ± 2.50 | 0.9613 |
|  |  | 8 | 187.6 ± 2.1 | 189.4 ± 3.4 | 189.4 ± 2.5 | 188.0 ± 3.2 | 189.5 ± 3.9 | 0.33 ± 0.84 | 0.6949 | 0.29 ± 2.49 | 0.9074 |
| LDL cholesterol, mg/dL | 2226 | 5 | 102.5 ± 2.3 | 102.1 ± 3.3 | 104.8 ± 2.9 | 105.7 ± 3.3 | 109.1 ± 3.6 | 1.52 ± 0.93 | 0.1056 | 3.81 ± 3.03 | 0.2104 |
|  |  | 6 | 103.1 ± 2.1 | 103.2 ± 2.9 | 105.1 ± 2.9 | 105.7 ± 3.3 | 108.9 ± 3.7 | 1.28 ± 0.93 | 0.1719 | 3.37 ± 2.98 | 0.2598 |
|  |  | 7 | 103.1 ± 2.2 | 102.8 ± 2.9 | 104.8 ± 2.9 | 105.5 ± 3.3 | 108.8 ± 3.6 | 1.25 ± 0.93 | 0.1805 | 3.32 ± 2.97 | 0.2657 |
|  |  | 8 | 102.8 ± 2.2 | 102.0 ± 3.0 | 104.5 ± 2.9 | 105.7 ± 3.3 | 108.8 ± 3.5 | 1.40 ± 0.92 | 0.1318 | 3.64 ± 2.99 | 0.2259 |
| HDL cholesterol, mg/dL | 5157 | 5 | 46.7 ± 0.6 | 47.0 ± 0.8 | 47.5 ± 0.9 | 47.6 ± 0.9 | 47.3 ± 1.0 | 0.20 ± 0.22 | 0.3596 | 0.28 ± 0.61 | 0.6415 |
|  |  | 6 | 47.4 ± 0.6 | 47.6 ± 0.8 | 48.2 ± 0.9 | 48.0 ± 1.0 | 47.9 ± 1.0 | 0.17 ± 0.22 | 0.4381 | 0.18 ± 0.64 | 0.7784 |
|  |  | 7 | 47.4 ± 0.6 | 47.9 ± 0.8 | 48.3 ± 0.9 | 48.1 ± 1.0 | 47.9 ± 1.0 | 0.17 ± 0.22 | 0.4556 | 0.16 ± 0.63 | 0.8017 |
|  |  | 8 | 46.7 ± 0.6 | 47.1 ± 0.8 | 47.6 ± 0.9 | 47.7 ± 0.9 | 47.3 ± 1.0 | 0.20 ± 0.22 | 0.3554 | 0.25 ± 0.61 | 0.6752 |
| Triglycerides, mg/dL^1^ | 2316 | 5 | 179.1 ±13.2 | 174.8 ±12.6 | 186.3 ±14.4 | 176.5 ±14.6 | 186.8 ±18.5 | 1.36 ± 3.65 | 0.8082 | 25.75 ± 20.35 | 0.2081 |
|  |  | 6 | 175.6 ± 12.7 | 171.4 ± 12.4 | 184.0 ± 14.0 | 174.6 ± 14.4 | 183.4 ± 18.3 | 1.56 ± 3.67 | 0.7682 | 24.85 ± 20.13 | 0.2511 |
|  |  | 7 | 175.7 ± 12.6 | 171.6 ± 12.5 | 184.2 ± 14.0 | 174.8 ± 14.3 | 183.5 ± 18.4 | 1.58 ± 3.69 | 0.8085 | 24.85 ± 20.10 | 0.2574 |
|  |  | 8 | 178.5 ± 13.1 | 174.7 ± 12.8 | 187.2 ± 14.3 | 177.4 ± 14.8 | 186.5 ± 18.5 | 1.57 ± 3.66 | 0.7962 | 25.71 ± 20.49 | 0.2445 |
| Waist circumference, cm | 5099 | 6 | 105.8 ± 0.8 | 105.0 ± 0.9 | 105.3 ± 1.1 | 106.0 ± 1.2 | 106.7 ± 1.2 | 0.20 ± 0.23 | 0.4004 | 1.20 ± 0.70 | 0.0883 |
|  |  | 7 | 105.8 ± 0.8 | 105.0 ± 0.9 | 105.3 ± 1.1 | 106.0 ± 1.2 | 106.7 ± 1.2 | 0.19 ± 0.23 | 0.4085 | 1.19 ± 0.69 | 0.0889 |

Values are least squares means ± SE. Model 5 is adjusted for age, gender, ethnicity, physical activity, poverty-to-income ratio, smoking status, alcohol intake, energy intake, saturated fat intake, sodium intake, carbohydrate intake, and BMI. Model 6 is adjusted for age, gender, ethnicity, physical activity, poverty-to-income ratio, smoking status, alcohol intake, energy intake, and energy-adjusted saturated fat and sodium intake (i.e., per 1000 kcal). Model 7 is further adjusted for energy adjusted carbohydrate intake (i.e., per 1000 kcal). Model 8 is further adjusted for BMI. HOMA-IR = fasting insulin (µU/mL) x fasting glucose (mmol/L)/22.5. Significance was set at P<0.01. ^1^Indicates variable was log-transformed to generate p-values; means, SE, and beta values are derived from the non-transformed model.

**Supplemental Table 7.** HEI-2020 component and total scores by diabetes status, most consumed potato preparation method and dietary pattern cluster in US adults: NHANES 2001–2018.

|  | Baked/Boiled | | | | Chips | | | | Fried | | | | Mashed | | | |
| --- | --- | --- | --- | --- | --- | --- | --- | --- | --- | --- | --- | --- | --- | --- | --- | --- |
|  | C0 | C1 | C2 | P | C0 | C1 | C2 | P | C0 | C1 | C2 | P | C0 | C1 | C2 | P |
| No diabetes  n | 24166 | 988 | 469 |  | 24166 | 2635 | 1371 |  | 24166 | 3411 | 2577 |  | 24166 | 1051 | 2437 |  |
| Total vegetables | 3.09 (0.02) | 4.03 (0.06)* | 4.13 (0.07)* | **<0.0001** | 3.07  (0.02) | 3.31 (0.04)* | 3.65 (0.05)* | **<0.0001** | 3.06 (0.02) | 3.33 (0.03)* | 3.29 (0.05)* | **<0.0001** | 3.08 (0.02) | 3.97 (0.06)* | 3.82 (0.04)* | **<0.0001** |
| Greens and beans | 2.04  (0.03) | 1.94 (0.12) | 2.18 (0.16) | 0.4774 | 2.02  (0.03) | 1.47 (0.06)* | 1.70 (0.09)* | **<0.0001** | 2.02 (0.03) | 1.44 (0.05)* | 1.51 (0.08)* | **<0.0001** | 2.03 (0.03) | 1.85 (0.11) | 1.63 (0.06)* | **<0.0001** |
| Total fruit | 2.43 (0.02) | 2.65 (0.09) | 2.12 (0.13) | **0.0055** | 2.41  (0.02) | 2.30 (0.06) | 1.99 (0.08)* | **<0.0001** | 2.40 (0.02) | 1.79 (0.05)* | 2.05 (0.06)* | **<0.0001** | 2.43 (0.02) | 1.93 (0.08)* | 2.32 (0.06) | **<0.0001** |
| Whole fruit | 2.67 (0.03) | 2.86 (0.09) | 2.45 (0.14) | 0.0382 | 2.64 (0.03) | 2.51 (0.07) | 2.27 (0.09)* | **0.0004** | 2.62 (0.03) | 2.00 (0.05)* | 2.29 (0.06)* | **<0.0001** | 2.67 (0.03) | 2.27 (0.10)* | 2.58 (0.07) | **0.0007** |
| Whole grains | 2.76 (0.04) | 2.60 (0.15) | 2.11 (0.17)* | **0.0013** | 2.74 (0.04) | 2.28 (0.06)* | 2.29 (0.10)* | **<0.0001** | 2.72 (0.04) | 1.93 (0.07)* | 2.05 (0.09)* | **<0.0001** | 2.77 (0.04) | 2.11 (0.13)* | 2.15 (0.08)* | **<0.0001** |
| Dairy | 5.40 (0.04) | 4.59 (0.15)* | 5.39 (0.20) | **<0.0001** | 5.38 (0.04) | 4.85 (0.11)* | 5.39 (0.11) | **<0.0001** | 5.39 (0.04) | 4.96 (0.08)* | 4.66 (0.11)* | **<0.0001** | 5.40 (0.04) | 5.15 (0.13) | 4.91 (0.10)* | **0.0001** |
| Total protein foods | 4.36 (0.01) | 4.52 (0.05)* | 4.59 (0.05)* | **<0.0001** | 4.36 (0.01) | 4.31 (0.03) | 4.50 (0.04)* | **0.0002** | 4.36 (0.01) | 4.40 (0.03) | 4.45 (0.03) | 0.0132 | 4.36 (0.01) | 4.65 (0.03)* | 4.47 (0.03)* | **<0.0001** |
| Seafood and plant protein | 2.85 (0.03) | 2.89 (0.11) | 2.70 (0.16) | 0.6076 | 2.84 (0.03) | 2.56 (0.07)* | 2.51 (0.10)* | **<0.0001** | 2.83 (0.03) | 2.31 (0.06)* | 2.48 (0.08)* | **<0.0001** | 2.85 (0.03) | 2.55 (0.11)* | 2.28 (0.07)* | **<0.0001** |
| Fatty acid ratio | 4.80 (0.04) | 4.81 (0.18) | 4.24 (0.23) | 0.0484 | 4.79 (0.04) | 5.17 (0.11)* | 4.86 (0.13) | **0.0062** | 4.78 (0.04) | 5.14 (0.08)* | 5.62 (0.11)* | **<0.0001** | 4.79 (0.04) | 4.29 (0.16)* | 4.57 (0.12) | **0.0058** |
| Sodium | 4.32 (0.04) | 5.17 (0.16)* | 3.81 (0.23) | **<0.0001** | 4.32 (0.04) | 4.96 (0.09)* | 3.29 (0.12)* | **<0.0001** | 4.32 (0.04) | 4.72 (0.09)* | 5.04 (0.10)* | **<0.0001** | 4.32 (0.04) | 3.22 (0.14)* | 4.55 (0.11) | **<0.0001** |
| Refined grain | 5.86 (0.04) | 7.57 (0.14)* | 6.60 (0.20)* | **<0.0001** | 5.87 (0.04) | 6.97 (0.10)* | 5.16 (0.14)* | **<0.0001** | 5.84 (0.04) | 6.44 (0.08)* | 7.16 (0.10)* | **<0.0001** | 5.88 (0.04) | 6.32 (0.14)* | 7.19 (0.09)* | **<0.0001** |
| Saturated fat | 6.06 (0.04) | 6.36 (0.14) | 5.60 (0.20) | **0.0051** | 6.05 (0.04) | 6.04 (0.11) | 5.15 (0.14)* | **<0.0001** | 6.06 (0.04) | 5.53 (0.09)* | 5.81 (0.10) | **<0.0001** | 6.05 (0.04) | 4.91 (0.16)* | 5.88 (0.09) | **<0.0001** |
| Added sugar | 6.69 (0.04) | 6.74 (0.14) | 6.83 (0.24) | 0.7869 | 6.66 (0.04) | 5.80 (0.11)* | 7.14 (0.13)* | **<0.0001** | 6.64 (0.04) | 5.93 (0.11)* | 6.43 (0.09) | **<0.0001** | 6.68 (0.04) | 7.27 (0.14)* | 6.04 (0.13)* | **<0.0001** |
| HEI-2020 total score | 53.3 (0.2) | 56.7 (0.7)* | 52.7 (1.0) | **<0.0001** | 53.2 (0.2) | 52.5 (0.4) | 49.9 (0.5)* | **<0.0001** | 53.0 (0.2) | 49.9 (0.3)* | 52.8 (0.4) | **<0.0001** | 53.3 (0.2) | 50.5 (0.6)* | 52.4 (0.4) | **<0.0001** |
| Diagnosed with diabetes  n | 3481 | 38 | 210 |  | 3481 | 255 | 247 |  | 3481 | 493 | 223 |  | 3481 | 353 | 167 |  |
| Total vegetables | 3.22 (0.05) | 4.04 (0.27)* | 4.26 (0.11)* | **<0.0001** | 3.21 (0.05) | 3.75 (0.15)* | 3.56 (0.13) | **0.0010** | 3.22 (0.05) | 3.58 (0.10)* | 3.60 (0.15) | **0.0034** | 3.21 (0.05) | 3.96 (0.10)* | 4.14 (0.11)* | **<0.0001** |
| Greens and beans | 2.00 (0.06) | 1.89 (0.50) | 1.43 (0.18) | 0.0195 | 2.00 (0.07) | 1.53 (0.17) | 1.44 (0.18)* | **0.0019** | 2.00 (0.07) | 1.38 (0.13)* | 1.60 (0.23) | **0.0001** | 1.99 (0.07) | 1.71 (0.17) | 1.93 (0.24) | 0.2526 |
| Total fruit | 2.57 (0.06) | 1.59 (0.21)* | 2.82 (0.20) | **<0.0001** | 2.55 (0.06) | 2.48 (0.23) | 2.52 (0.19) | 0.9496 | 2.55 (0.06) | 2.07 (0.14)* | 1.94 (0.20)* | **<0.0001** | 2.56 (0.06) | 2.59 (0.16) | 2.39 (0.21) | 0.6897 |
| Whole fruit | 2.90 (0.07) | 2.01 (0.30)* | 3.35 (0.21) | **0.0014** | 2.88 (0.07) | 2.70 (0.25) | 2.68 (0.20) | 0.5238 | 2.88 (0.07) | 2.53 (0.17) | 2.19 (0.20)* | **0.0002** | 2.90 (0.07) | 2.88 (0.17) | 2.80 (0.27) | 0.9478 |
| Whole grains | 3.41 (0.09) | 3.32 (0.56) | 3.03 (0.29) | 0.4628 | 3.39 (0.10) | 3.53 (0.40) | 3.12 (0.33) | 0.6682 | 3.39 (0.09) | 2.63 (0.21)* | 2.10 (0.26)* | **<0.0001** | 3.42 (0.10) | 3.39 (0.24) | 2.61 (0.33) | 0.0628 |
| Dairy | 5.22 (0.11) | 6.27 (0.81) | 4.64 (0.27) | 0.0375 | 5.22 (0.11) | 5.56 (0.24) | 4.57 (0.27) | 0.0171 | 5.23 (0.11) | 4.28 (0.22)* | 5.08 (0.34) | **0.0015** | 5.24 (0.11) | 5.00 (0.21) | 4.59 (0.34) | 0.1479 |
| Total protein foods | 4.52 (0.03) | 4.56 (0.21) | 4.72 (0.06) | 0.0324 | 4.53 (0.03) | 4.26 (0.15) | 4.75 (0.06)* | **0.0019** | 4.53 (0.03) | 4.69 (0.05) | 4.53 (0.08) | 0.0107 | 4.52 (0.03) | 4.69 (0.09) | 4.72 (0.07) | 0.0197 |
| Seafood and plant protein | 2.90 (0.06) | 2.23 (0.50) | 3.02 (0.21) | 0.3617 | 2.90 (0.06) | 2.55 (0.20) | 2.74 (0.20) | 0.1781 | 2.91 (0.06) | 2.59 (0.17) | 2.52 (0.24) | 0.1030 | 2.90 (0.06) | 2.87 (0.18) | 3.05 (0.22) | 0.7965 |
| Fatty acid ratio | 5.04 (0.10) | 3.76 (0.69) | 5.23 (0.35) | 0.1456 | 5.04 (0.10) | 5.59 (0.29) | 6.20  (0.27)* | **0.0003** | 5.03 (0.10) | 6.41 (0.22)* | 5.34 (0.34) | **<0.0001** | 5.02 (0.10) | 4.98 (0.28) | 4.63 (0.29) | 0.4758 |
| Sodium | 3.39 (0.08) | 3.62 (0.73) | 3.92 (0.32) | 0.2169 | 3.38 (0.08) | 3.66 (0.31) | 3.51 (0.26) | 0.6408 | 3.37 (0.08) | 3.56 (0.23) | 3.45 (0.33) | 0.6893 | 3.38 (0.08) | 3.29 (0.27) | 2.85 (0.34) | 0.2849 |
| Refined grain | 5.56 (0.10) | 5.98 (0.75) | 6.72 (0.36)* | **0.0081** | 5.57 (0.10) | 5.70 (0.45) | 6.11 (0.26) | 0.1406 | 5.57 (0.10) | 6.63 (0.26)* | 5.94 (0.29) | **0.0013** | 5.59 (0.10) | 7.49 (0.19)* | 6.61 (0.32)* | **<0.0001** |
| Saturated fat | 5.68 (0.10) | 4.83 (0.85) | 6.41 (0.34) | 0.0787 | 5.66 (0.10) | 5.53 (0.27) | 5.92 (0.26) | 0.5380 | 5.65 (0.10) | 5.35 (0.23) | 5.00 (0.29) | 0.0668 | 5.64 (0.10) | 5.44 (0.26) | 5.22 (0.32) | 0.3690 |
| Added sugar | 7.89 (0.07) | 8.51 (0.66) | 8.03 (0.18) | 0.4787 | 7.88 (0.07) | 7.88 (0.24) | 8.09 (0.22) | 0.6336 | 7.89 (0.07) | 7.88 (0.18) | 7.28 (0.36) | 0.2516 | 7.89 (0.07) | 7.49 (0.28) | 7.28 (0.44) | 0.1085 |
| HEI-2020 total score | 54.3 (0.4) | 52.6 (3.2) | 57.6 (1.1) | 0.0170 | 54.2 (0.4) | 54.7 (1.6) | 55.2 (1.3) | 0.7423 | 54.2 (0.4) | 53.6 (0.9) | 50.6 (1.5) | 0.0608 | 54.3 (0.4) | 55.8 (0.8) | 52.8 (1.4) | 0.1514 |

Values are least squares means ± SE. Variables were adjusted for age, gender, ethnicity, physical activity, PIR ratio, smoking status, alcohol intake. Significance was set at P<0.01. *P<0.01 compared to cluster 0. Abbreviations: C0, cluster 0; C1, cluster 1; C2, cluster 2.

**Supplemental Table 8.** Use of glucose, blood pressure, and lipid lowering medications in US adults categorized by diabetes status: NHANES 2001-2018.

|  | No diabetes | | Diagnosed with diabetes | |
| --- | --- | --- | --- | --- |
|  | n | Yes (%) | n | Yes (%) |
| **Glucose lowering medication** |  |  |  |  |
| Any | 425 | 0.816 ± 0.061 | 4460 | 81.3 ± 0.7 |
| Insulin | 12 | 0.016 ± 0.005 | 1295 | 24.0 ± 0.8 |
| Biguanide (metformin) | 322 | 0.661 ± 0.060 | 2844 | 53.7 ± 1.0 |
| Sulfonylurea | 110 | 0.144 ± 0.019 | 1851 | 31.2 ± 1.0 |
| Thiazolidinedione | 40 | 0.064 ± 0.014 | 597 | 11.5 ± 0.6 |
| Dipeptidyl peptidase 4 inhibitor | 21 | 0.026 ± 0.007 | 349 | 7.2 ± 0.5 |
| Sodium-glucose cotransporter-2 inhibitor | 2 | 0.002 ± 0.001 | 52 | 1.3 ± 0.3 |
| Glucose like peptide-1 receptor agonist | 2 | 0.011 ± 0.008 | 96 | 2.4 ± 0.3 |
| **Blood pressure lowering medication** |  |  |  |  |
| Any | 9718 | 21.3 ± 0.4 | 4033 | 71.8 ± 0.9 |
| Angiotensin converting enzyme inhibitor | 3582 | 7.8 ± 0.2 | 2218 | 40.3 ± 0.9 |
| Angiotensin II inhibitor | 2035 | 4.6 ± 0.2 | 1023 | 18.0 ± 0.7 |
| Diuretic | 4343 | 9.3 ± 0.2 | 1817 | 30.8 ± 0.8 |
| Calcium channel blocking agent | 2879 | 5.3 ± 0.2 | 1201 | 19.5 ± 0.7 |
| Beta-adrenergic blocker agent | 3795 | 8.4 ± 0.2 | 1587 | 28.5 ± 0.9 |
| **Lipid lowering medication** |  |  |  |  |
| Statin | 4945 | 11.6 ± 0.3 | 2763 | 51.3 ± 1.0 |

Values are means ± SE.
